# Lignin Biosynthesis Driven by *CAD* Genes Underpins Drought Tolerance in Sugarcane: Genomic Insights for Crop Improvement

**DOI:** 10.3390/plants14172735

**Published:** 2025-09-02

**Authors:** Yue Wang, Weitong Zhao, Peiting Li, Junjie Zhao, Zhiwei Yang, Chaohua Huang, Guoqiang Huang, Liangnian Xu, Jiayong Liu, Yong Zhao, Yuebin Zhang, Zuhu Deng, Xinwang Zhao

**Affiliations:** 1 National Engineering Research Center for Sugarcane, Fujian Agriculture and Forestry University, Fuzhou 350002, China; 15735004297@163.com (Y.W.); zhaowt0810@163.com (W.Z.); lipeiting147258@163.com (P.L.); wlb051321@163.com (J.Z.); 19556907686@163.com (Z.Y.); ganzhe403@163.com (C.H.); hgq94@163.com (G.H.); xuliangnian@163.com (L.X.); zhaoyong@yaas.org.cn (Y.Z.); 2 Yunnan Key Laboratory of Sugarcane Genetic Improvement, Sugarcane Research Institute, Yunnan Academy of Agricultural Sciences, Kaiyuan 661600, China; liujiayong@yaas.org.cn (J.L.); ynzyb@sohu.com (Y.Z.); 3 Guangxi Key Laboratory for Sugarcane Biology, Guangxi University, Nanning 530005, China

**Keywords:** *Saccharum* spp., drought resistance, lignin precursor regulation, *CAD* phylogenetic clades, molecular breeding

## Abstract

Sugarcane (*Saccharum* spp.), a vital economic crop, suffers significant yield losses from drought. This study elucidates the genetic regulation of lignin biosynthesis—a key drought-resistance mechanism—by analyzing three contrasting accessions: drought-sensitive *Saccharum officinarum* (Badila), drought-resistant hybrid (XTT22), and drought-tolerant wild *Saccharum spontaneum* (SES-208) under progressive drought (7–21 days). Physiological analyses revealed pronounced lignin accumulation in XTT22 roots/leaves, driven by elevated coniferyl/sinapyl alcohol substrates, while Badila showed minimal deposition. Genomic characterization of cinnamyl/sinapyl alcohol dehydrogenase (*CAD/SAD*) families across six sugarcane genomes identified 322 genes phylogenetically clustered into three clades. Class I members (*CAD1*, *CAD5*, etc.) were critical for lignin biosynthesis, with tandem/segmental duplications driving family expansion and promoters enriched in stress-responsive *cis*-elements (ABA, MeJA, light). Transcriptomics and qRT-PCR confirmed strong correlations between Class I *CAD* expression, lignin content, and drought tolerance. These findings establish *CAD* Class I genes as novel molecular targets for enhancing drought resilience in sugarcane breeding programs.

## 1. Introduction

Sugarcane (*Saccharum* spp.) is a globally vital crop for sugar production and bioenergy. According to the Food and Agriculture Organization (FAO) and the Organisation for Economic Co-operation and Development (OECD), sugarcane contributes over 85% of the world’s sugar supply [1]. As a typical C4 plant within the Poaceae family, sugarcane requires substantial water throughout its entire growth cycle, spanning from germination, root establishment, tillering, stem elongation, sucrose accumulation, to maturation [2]. In the context of escalating global environmental challenges, sustainable sugarcane production necessitates continuous crop improvement. Climate change is projected to exacerbate extreme weather events, including frost, heatwaves, droughts, and floods [3,4]. Over 80% of sugarcane is cultivated in tropical and subtropical regions—such as Brazil, India, and China—where erratic precipitation patterns persistently threaten crop productivity through recurrent drought stress [5]. Furthermore, sugarcane’s prolonged growth cycle (12–18 months) renders it vulnerable to seasonal and extended drought periods [6,7]. Therefore, it is of great significance to study the drought-resistant mechanism of sugarcane and to select and breed more drought-resistant sugarcane varieties.

Lignin is an amorphous aromatic heteropolymer ubiquitously present in plants, composed of oxygenated phenylpropanoid derivatives such as coniferyl alcohol, sinapyl alcohol, and p-coumaryl alcohol [8]. As a key component of cell walls and mechanical tissues, lignin predominantly accumulates in the secondary cell walls of structural tissues (e.g., fibers, sclerenchyma), protective tissues (e.g., epidermis), and vascular tissues (e.g., xylem vessels or tracheids) [9]. It plays pivotal roles in maintaining cell wall integrity, stem rigidity, water transport, mechanical support, and defense against pathogens [10]. Lignin forms a complex cross-linked network with cellulose and hemicellulose in the cell wall, enhancing mechanical strength, while its hydrophobic properties minimize water loss and facilitate efficient transport of water, minerals, and organic compounds through xylem and phloem [11]. In vascular tissues, lignified cell walls enable roots to absorb water and provide structural support. Additionally, lignin-cellulose interactions establish a physical barrier against pathogen invasion, serving as a critical defense mechanism [12].

Lignin biosynthesis is intricately linked to plant development and stress adaptation. Under abiotic stress, lignin content in cell walls undergoes dynamic modulation. For instance, drought stress induces lignin accumulation in *Caragana korshinskii* leaves under both laboratory and field conditions [13]. Genetic studies reveal that *OsNAC5* activates cinnamoyl-CoA reductase (*CCR*) to promote lignin deposition in rice roots, thereby enhancing drought tolerance [14]. Similarly, overexpression of *VlbZIP30* in grapevines elevates leaf lignification and improves drought resistance [15]. These findings underscore lignin’s dual role in structural reinforcement and stress resilience, positioning it as a strategic target for crop improvement.

Lignin monomers are synthesized through the phenylpropanoid pathway, starting with the deamination of phenylalanine (or tyrosine) to form cinnamic acid. This precursor undergoes sequential hydroxylation, methylation, and reduction reactions, ultimately yielding three primary monolignols: coumaryl alcohol, coniferyl alcohol, and sinapyl alcohol. Based on structural differences among these monomers, lignin is classified into three types: syringyl lignin (S-lignin), polymerized from syringyl propane units; guaiacyl lignin (G-lignin), composed of guaiacyl propane units; and p-hydroxyphenyl lignin (H-lignin), formed by p-hydroxyphenyl propane units [16,17].

The Phenylpropionic acid pathway, a universal secondary metabolic pathway in plants, involves 14 catalytic enzymes, including tyrosine ammonia-lyase (*TAL*), phenylalanine ammonia-lyase (*PAL*), cinnamate 4-hydroxylase (*C4H*), coumarate 3-hydroxylase (*C3H*), ferulate 5-hydroxylase (*F5H*), 4-coumarate-CoA ligase (*4CL*), caffeoyl-CoA O-methyltransferase (*CCoAOMT*), coumaroyl-CoA hydroxylase (*CCH*), caffeoylshikimate transferase (*CQT*), caffeoyl-CoA shikimate transferase (*CST*), caffeic acid O-methyltransferase (*COMT*), cinnamoyl-CoA reductase (*CCR*), cinnamyl alcohol dehydrogenase (*CAD*), and sinapyl alcohol dehydrogenase (*SAD*). Among these, cinnamyl alcohol dehydrogenase (*CAD*) catalyzes the final step in monolignol biosynthesis, converting cinnamyl aldehydes into their corresponding alcohols. This Zn^2+^ and NADPH-dependent enzyme belongs to the medium-chain dehydrogenase/reductase (MDR) superfamily [18,19]. The *CAD* gene was first identified in tobacco stems [20], and subsequent studies have revealed the evolution, origin, and classification of the *CAD/SAD* gene family across plant species, with *SAD* classified as a member of the Class II subfamily of *CAD* genes [21]. Functional studies in plants such as strawberry (*Fragaria* spp.) suggest that *CAD* genes may regulate fruit firmness [22], while in poplar (*Populus*), Class I *CAD* genes exhibit elevated expression in lignified tissues, highlighting their role in xylem development [23].

In plants, *CAD* genes are highly homologous. Phylogenetic analyses have classified *CAD* genes into three major classes: Class I *CADs*, which play an important role in lignin synthesis and are mainly involved in the coniferyl alcohol and sinapyl alcohol [24]. Nine *CAD* genes were identified in Arabidopsis thaliana, among which *AtCAD4* and *AtCAD5* are considered true *CAD* in Class I [25,26,27]. The Class II consists of members that are more similar to the sinapyl alcohol dehydrogenase (*SAD*), and they will not be conserved as much as the true *CAD*, *AtCAD2*, *AtCAD3*, *AtCAD7*, and *AtCAD8* in Arabidopsis thaliana have higher homology to the mustard alcohol dehydrogenase *SAD*, with *AtCAD6* and *AtCAD9* being Class II. The Class III is mainly found in terrestrial plants, which may not be responsible for the biosynthesis of lignin, but has an important function in plant development and is necessary for the adaptation of terrestrial plants. For example, *AtCAD1* in Arabidopsis thaliana does not show high activity for lignin synthesis; it is a Class III [28].

To address the critical challenge of drought-induced yield loss in sugarcane (*Saccharum* spp.), understanding the molecular mechanisms underlying lignin-mediated drought resistance is essential. Previous studies have demonstrated that lignin accumulation enhances plant stress tolerance, yet the dynamic regulation of lignin biosynthesis and its metabolic precursors during drought adaptation remains poorly characterized in sugarcane [11]. Notably, variations in lignin deposition patterns between drought-resistant and sensitive genotypes, particularly in roots and leaves under progressive drought stress, could reveal key adaptive strategies. Furthermore, while cinnamyl alcohol dehydrogenase (*CAD*) and sinapyl alcohol dehydrogenase (*SAD*) gene families are known to drive lignin biosynthesis, their genomic organization, evolutionary expansion, and stress-responsive regulation in sugarcane have not been systematically investigated across diverse germplasm [21]. Integrating phenotypic analyses of lignin metabolism with comparative genomics of *CAD/SAD* gene families in representative sugarcane genomes could provide novel insights into the genetic basis of drought tolerance, bridging the gap between metabolic adaptation and molecular breeding targets.

## 2. Materials and Methods

Modern sugarcane cultivars derive their genetic composition from two primary ancestral lineages: *S. officinarum *(*Saccharum officinarum*) (tropical noble cane), contributing approximately 75% of the genome and serving as the genetic foundation for high sugar content and yield, and *S. spontaneum* (*Saccharum spontaneum*) (wild cane), which provides disease and stress resistance genes and accounts for about 15% of the cultivated genome [29]. This study utilized three materials—Badila (*S. officinarum*), SES-208 (*S. spontaneum*), and XTT22 (*S. hybrid*) (*Saccharum hybrid*)—to investigate drought response mechanisms. Phenotypic observations were conducted across varying drought stages, with lignin content quantified in leaves and roots under drought stress. Additionally, lignin-related small molecules in XTT22 were analyzed before and after drought to compare metabolic differences between roots and leaves during drought adaptation. To further explore the role of lignin metabolism in drought resistance, systematic bioinformatics analyses were performed on *CAD* (cinnamyl alcohol dehydrogenase) and *SAD* (sinapyl alcohol dehydrogenase) gene families across multiple sugarcane reference genomes, including *Erianthus rufipilus*, *S. spontaneum* AP85-441, *S. spontaneum* Np-X, *S. officinarum* LA-Purple, *S. hybrid* R570 and *S. hybrid* XTT22. Gene expression profiles of *CAD* under diverse stress conditions were also examined. These integrated approaches aim to elucidate the functional contributions of lignin metabolites, associated small molecules, and key genes in enhancing drought tolerance in sugarcane roots and leaves.

### 2.1. Plant Materials and Growth Conditions

Sugarcane seedlings of SES-208, Badila, and XTT22 were cultivated in pots at the sugarcane greenhouse of Fujian Agriculture and Forestry University, with a growth period of 100 days under daily morning and evening watering.

### 2.2. Drought Simulation Experiment

The experiment used potted plants, with pots measuring 17.2 cm in diameter and 17 cm in height. The drought stress treatment was initiated in the form of stopping watering when the sugarcane grew to the early stage of elongation. The experiment was set up with two groups: control and treatment. Control group: normal watering to ensure vigorous growth of plants; treatment group: drought stress with no watering. Plants were cultivated under optimal conditions for 100 days before initiating drought stress by water withholding. The drought stress experiment was divided into 4 stages: one sample was taken before stopping water supply; one sample was taken 7 days after stopping water supply, when the cane leaves curled up, some leaves began to wither, and there was no ‘spitting water’ in the morning; and one sample each was taken 14 days and 21 days after stopping water supply. Set up 3 biological replicates per sample. Sampling was carried out 4 times in total, at 6:00~7.00 am.

### 2.3. Lignin Content Quantification

Lignin levels were measured using a microassay with the Solarbio Lignin Content Detection Kit (Beijing, China), with three biological replicates per sample. Fresh leaves and roots were cleaned, oven-dried at 80 °C to constant weight, ground into powder under liquid nitrogen, sieved through a 30 mesh screen, and 3 mg aliquots were transferred to 1.5 mL tubes for analysis. Procedures followed the manufacturer’s protocol. Lignin content (mg/g) was calculated using the formula Lignin = 2.1842 × ΔA/W, where ΔA represents absorbance difference, W is sample weight (g), and constants include extinction coefficient (ξ = 23.35 mL/mg/cm), cuvette pathlength (d = 1 cm), acetylation reaction volume (0.612 mL), supernatant volume (0.012 mL), and detection volume (0.6 mL).

### 2.4. Metabolite Profiling in the Lignin Pathway

Small-molecule metabolites in XTT22 samples under normal irrigation (14 days) and drought stress (14 days) were analyzed. The experiment was performed with three biological replicates. Fresh plant tissues were freeze-dried under vacuum, homogenized to powder using a grinding mill, and 100 mg aliquots were dissolved in 0.6 mL extraction solvent (Metware, Wuhan, China). After overnight incubation at 4 °C with six intermittent vortex-mixing cycles to enhance extraction efficiency, samples were centrifuged at 10,000× *g* for 10 min. The supernatant was filtered through a 0.22-μm microporous membrane and stored in injection vials for UPLC-MS/MS analysis [30].

Metabolite identification was performed by matching primary and secondary mass spectra with public databases, including MassBank (https://massbank.eu/ (accessed on 27 October 2023)), KNApSAcK (http://kanaya.naist.jp/KNApSAcK/ (accessed on 27 October 2023)), HMDB (http://www.hmdb.ca/ (accessed on 27 October 2023)), and METLIN (https://metlin.scripps.edu/ (accessed on 27 October 2023)) [31]. Isotopic signals, adducts (K^+^, Na^+^, NH_4_^+^), and fragment ions from higher molecular weight compounds were excluded during annotation. Quantitative analysis was conducted using multiple reaction monitoring (MRM) mode on a triple quadrupole mass spectrometer. Peak areas of metabolites were integrated, and cross-sample normalization was applied to correct variations in signal intensity [32]. In this study, inter-group differential analysis was performed using the Wilcoxon rank-sum test and fold change (FC) values. Screening criteria: Metabolites with FC ≥ 2 and FC ≤ 0.5 were selected as the final differentially expressed metabolites. Fold change represents the ratio of expression levels between two groups.

### 2.5. Identification of CAD Gene Family Members and Physicochemical Property Prediction

The sugarcane genome data were obtained from the Sugarcane Genome Database (https://sugarcane.gxu.edu.cn/scdb/ (accessed on 10 May 2024)). To identify *CAD* family members, Arabidopsis *CAD* protein sequences were downloaded from NCBI and used for homology-based alignment to extract putative sugarcane *CAD* protein sequences. Candidate sequences were annotated via NCBI (https://www.ncbi.nlm.nih.gov/ (accessed on 15 May 2024)) BLAST and manually curated. Additionally, the HMM profiles of *CAD* domains (PF08240 and PF00107) were retrieved from the Pfam database (https://www.ebi.ac.uk/interpro/entry/pfam/#table (accessed on 15 May 2024)) and applied to screen sugarcane *CAD* members using TBtools-II v2.154. The intersection of results from both methods was used to define the final *CAD* gene family. Physicochemical properties of proteins were predicted using the ExPASy online tool (https://www.expasy.org/ (accessed on 17 October 2024)).

### 2.6. Prediction of Conserved Motifs, Gene Structures, Domains, and Cis-Acting Elements

Conserved motifs in sugarcane *CAD* proteins were predicted using MEME (https://meme-suite.org/meme/tools/meme (accessed on 27 June 2024)) with 10 motifs and default parameters. Exon-intron structures were analyzed using TBtools-II v2.154. Conserved domains were identified via Batch CD-Search, and incomplete genes were filtered out. For cis-acting element analysis, 2000 bp upstream promoter regions of sugarcane *CAD* genes were extracted using TBtools-II v2.154 and submitted to PlantCARE (https://bioinformatics.psb.ugent.be/webtools/plantcare/html/ (accessed on 23 September 2024)). Results were visualized with TBtools-II v2.154, with distinct elements color-coded.

### 2.7. Phylogenetic Tree Construction

Arabidopsis *CAD* protein sequences were retrieved from NCBI. Multiple sequence alignment was performed using ClustalW, and a Neighbor-Joining (NJ) phylogenetic tree was constructed with MEGA11, incorporating *CAD* members from sugarcane, Arabidopsis, Tobacco, Rice, and Sorghum. Bootstrap analysis was conducted with 1000 replicates. The tree was refined using EVOLVIEW (https://evolgenius.info//evolview-v2 (accessed on 28 October 2024)).

### 2.8. Chromosomal Localization, Gene Duplication, and Synteny Analysis

Chromosomal positions of sugarcane *CAD* genes were mapped using genome annotation files and visualized via TBtools-II v2.154 (Gene Location Visualize). Intraspecific and interspecific synteny analyses were performed using Circos and Dual Synteny Plot modules in TBtools-II v2.154.

### 2.9. Transcriptome Analysis

Drought-responsive expression patterns of 7 and 24 sugarcane *CAD* homologs were analyzed using RNA-seq data from roots (Badila, XTT22, and SES-208 under 5, 10, and 15 days of drought) and leaves (Badila and XTT22 under 4, 8, 16, and 32 h of drought). Heatmaps were generated using TBtools-II v2.154.

### 2.10. RNA Extraction and qRT-PCR Analysis

Total RNA was isolated using TRIzol reagent. cDNA synthesis and quantitative real-time PCR (qRT-PCR) were performed using a Beyotime fluorescence quantitative PCR kit (Beijing, China). The sugarcane *GAPDH* gene served as the internal control. Relative expression levels were calculated using the 2^−ΔΔCt^ method. Primer sequences are listed in Supplementary Table S1.

### 2.11. Statistical Analysis

Data were analyzed by one-way ANOVA. Figures were prepared using Adobe Illustrator CS6, and data organization was conducted in Microsoft^®^ Excel^®^ 2021 MSO. Statement: During the preparation of this work, the authors used Deepseek-V3 in order to polish the article. After using this service, the authors reviewed and edited the content as needed and take full responsibility for the content of the published article.

## 3. Results

### 3.1. Phenotypic Changes in Badila, XTT22, and SES-208 Under Drought Stress

**Figure 1:**
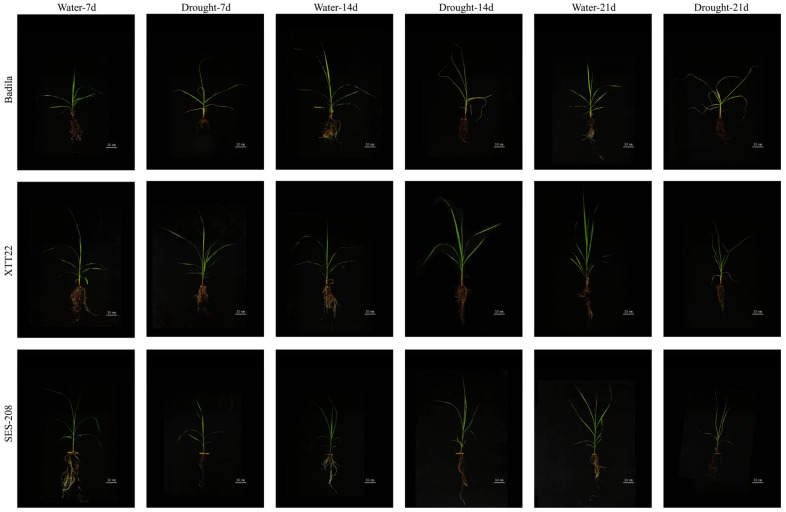
Phenotypes of Badila, XTT22, and SES-208 under drought stress. At 7, 14, and 21 days after drought treatment.

To investigate drought effects on sugarcane varieties and lignin content dynamics, Badila, XTT22, and SES-208 plants were subjected to drought treatment after 100 days of greenhouse cultivation (Figure 1). Badila exhibited the earliest stress symptoms. At 7 days of drought, Badila showed slight wilting in leaves and roots, while XTT22 and SES-208 remained phenotypically normal. By 14 days, Badila leaves were severely curled and yellowed, with increased root desiccation. At this stage, XTT22 and SES-208 began displaying mild water-deficit symptoms in leaves. After 21 days, Badila leaves were almost entirely necrotic, with roots hardened and blackened. XTT22 exhibited complete senescence of older leaves, curling of new leaves, and exacerbated root desiccation, though some white viable roots persisted. SES-208 was minimally affected by drought, showing only dehydrated and wilted leaves and reduced white vigor roots.

### 3.2. Drought Stress Alters Lignin Content in Sugarcane

**Figure 2:**
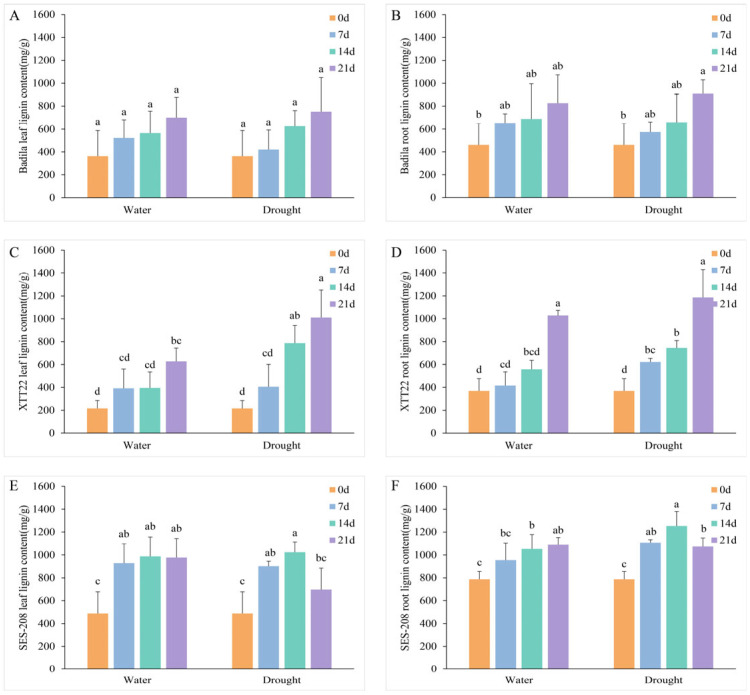
The lignin content in leaves and roots of three materials under drought and normal conditions. (**A**) Badila leaf lignin content (mg/g). (**B**) Badila root lignin content (mg/g). (**C**) XTT22 leaf lignin content (mg/g). (**D**) XTT22 root lignin content (mg/g). (**E**) SES-208 leaf lignin content (mg/g). (**F**) SES-208 root lignin content (mg/g). 0, 7, 14, and 21 days of drought treatment (0 d, 7 d, 14 d, 21 d). Data represent mean ± SD (*n* = 3). Error bars indicate standard deviation. Different lowercase letters denote significant differences (*p* < 0.05) compared to 0 DAD.

To investigate the impact of drought on lignin synthesis, lignin content was measured in leaves and roots of the three varieties (Figure 2).

There was no significant difference (*p* > 0.05) in the increase in lignin content in both leaves and roots of Badila (Figure 2A,B). There was also no significant difference (*p* > 0.05) between normal and drought treatments, which showed slight increase in lignin content with time, but no significant change from normal after drought stress. In leaves and roots of XTT22 (Figure 2C,D), lignin content increased in a stepwise manner under both normal and drought conditions, and it increased faster and higher under drought stress, and there was a significant difference (*p* < 0.05) between normal and drought groups in leaves and roots. Lignin content in leaves (Figure 2E) and roots (Figure 2F) of SES-208 increased significantly (*p* < 0.05) at 7 d in all treatments. There was no significant difference (*p* > 0.05) between normal and drought treatments in leaves, and a significant difference (*p* < 0.05) between normal and drought group treatments in roots. Its lignin content was significantly higher than Badila in all treatments.

Overall, the lignin content in Badila, XTT22, and SES-208 exhibited an increasing trend with prolonged drought exposure. SES-208 displayed the highest initial lignin content, which remained the highest after 7 days of drought treatment, while Badila consistently showed the lowest levels at both 7 and 14 days. By 21 days of drought, XTT22 accumulated the highest lignin content, whereas SES-208 experienced a significant decline, reaching levels comparable to Badila. Notably, XTT22 and SES-208 demonstrated greater fluctuations in lignin content across treatments and exhibited stronger drought tolerance compared to Badila. These findings suggest that lignin biosynthesis in sugarcane is closely associated with drought adaptation, with dynamic lignin accumulation potentially serving as a key mechanism for enhancing drought resistance.

### 3.3. XTT22 Exhibits Superior Drought Tolerance Compared to Badila at 14 DAD

**Figure 3:**
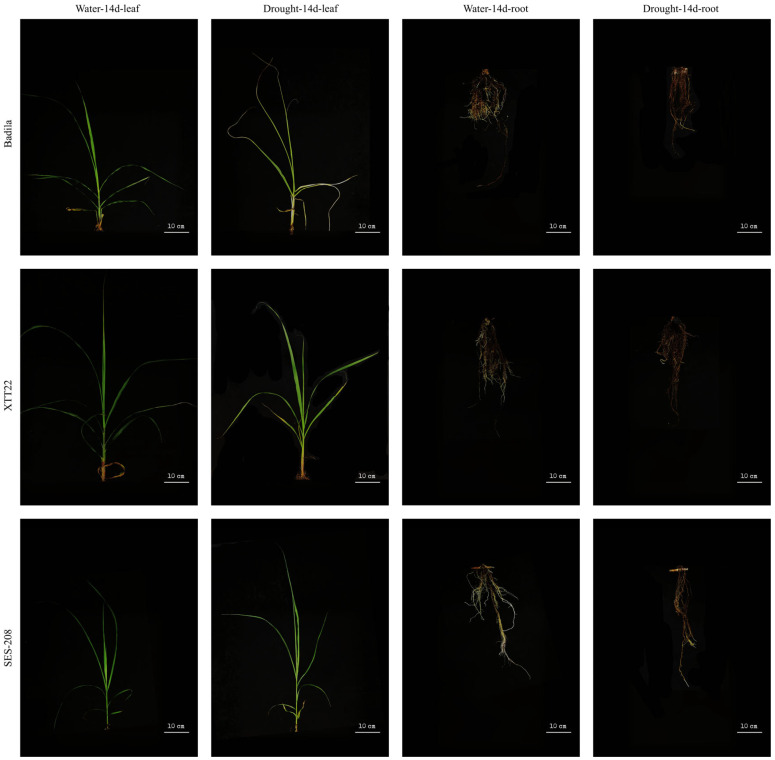
Phenotypic changes in leaves and roots of Badila, XTT22, and SES-208 after 14 days of normal watering and drought.

Measurements of lignin content in leaves and roots across the three varieties revealed significant variations in XTT22, with marked differences (*p* < 0.05) between control and drought conditions in both tissues. Significance analysis of XTT22 leaves and roots under control versus drought at 14 DAD yielded *p*-values of 0.031 and 0.034, respectively (both *p* < 0.05), prompting a focused examination of phenotypic changes at this stage (Figure 3). Phenotypic observations demonstrated that Badila displayed the poorest drought tolerance, characterized by low and stable lignin levels. SES-208 exhibited moderate drought resistance, with high baseline lignin content but minimal variation between control and drought conditions. In contrast, XTT22 showed robust drought tolerance, accompanied by substantial lignin accumulation under stress. These results indicate that lignin biosynthesis positively contributes to drought adaptation in sugarcane, and drought stress actively promotes lignin synthesis.

### 3.4. Drought Stress Enhances Lignin Synthesis via Accumulation of Coniferyl Alcohol and Sinapyl Alcohol

To elucidate the metabolomic regulation of lignin biosynthesis under drought, this study quantified 13 lignin-related metabolites in leaves and roots of XTT22 after 14 days of drought (Supplementary Figure S1A–C). In leaves, p-Coumaraldehyde, L-Phenylalanine, Sinapic acid, Coniferyl alcohol, p-Coumaric acid, Caffeate, Ferulic acid, Sinapyl alcohol, p-Coumaryl alcohol, 4-Hydroxy-3-methoxycinnamaldehyde, Cinnamic acid, and Caffeyl aldehyde exhibited increased levels compared to controls, with L-Phenylalanine showing the highest abundance, followed by p-Coumaric acid. In roots, Sinapic acid, Coniferyl alcohol, Caffeate, Ferulic acid, Sinapyl alcohol, and p-Coumaryl alcohol increased, while p-Coumaraldehyde, L-Phenylalanine, p-Coumaric acid, 4-Hydroxy-3-methoxycinnamaldehyde, Cinnamic acid, and Caffeyl aldehyde decreased. Notably, L-Phenylalanine remained the most abundant metabolite in roots, followed by p-Coumaric acid.

**Figure 4:**
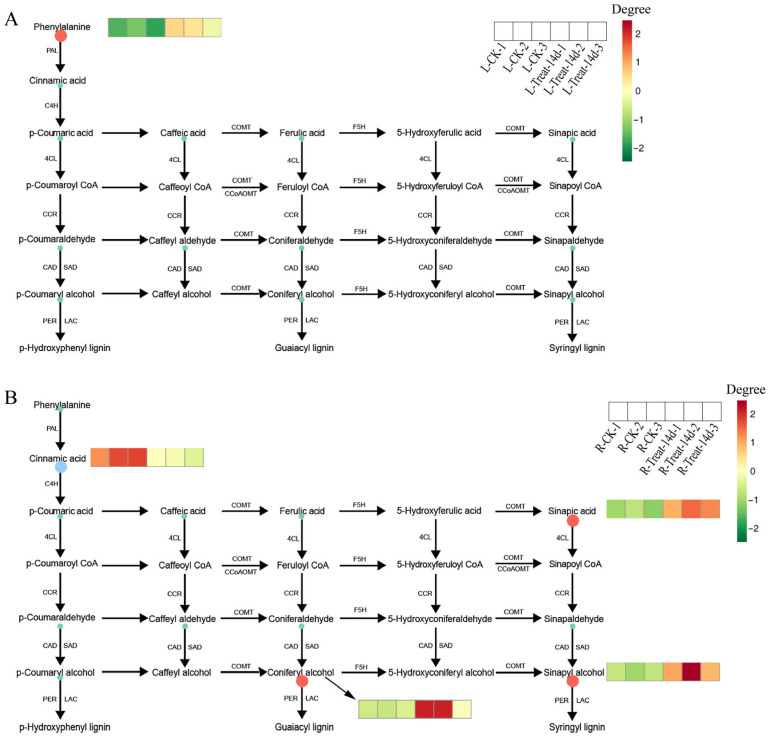
KEGG pathway annotation. (**A**) Leaf-CK_vs_Leaf-Treat-14d. (**B**) Root-CK_vs_Root-Treat-14d. CK: control group under normal growth; Treat: drought-treated group after 14 DAD. Orange indicates metabolites significantly upregulated in the treated group, green denotes metabolites detected without significant changes, and blue represents metabolites significantly downregulated.

Significance analysis and KEGG pathway annotation were performed to identify key drivers of lignin accumulation. In leaves, L-Phenylalanine levels increased significantly (*p* < 0.05) under drought (Figure 4A), suggesting its pivotal role in promoting lignin synthesis. No other metabolites showed significant changes in leaves. In roots, Sinapic acid, Coniferyl alcohol, and Sinapyl alcohol levels rose significantly (*p* < 0.05), while Cinnamic acid decreased markedly (Figure 4B). These findings highlight tissue-specific metabolic reprogramming, where drought-induced accumulation of Coniferyl alcohol and Sinapyl alcohol in roots and L-Phenylalanine in leaves collectively enhances lignin biosynthesis, reinforcing drought adaptation.

### 3.5. Identification of CAD Genes and Analysis of Protein Physicochemical Properties in Sugarcane

Metabolomics analyses showed that the small molecules Coniferyl alcohol and Sinapyl alcohol, which are the last step in the synthesis of lignin monomers, exhibited large differences before and after drought in sugarcane roots and leaves, and that two enzymes, cinnamyl alcohol dehydrogenase (*CAD*) and stigmasterol dehydrogenase (*SAD*), were the key enzymes for the synthesis of Coniferyl alcohol and Sinapyl alcohol, and Sinapyl alcohol. A total of 322 *CAD* genes were identified across six sugarcane-related genomes: *Erianthus rufipilus* (*EruCAD*, 13), *S. spontaneum* AP85-441 (*SsCAD*, 23), *S.spontaneum* Np-X (*NpCAD*, 39), *S. officinarum* LA-Purple (*SoCAD*, 70), *S. hybrid* R570 (*ShR570CAD*, 66), and *S. hybrid* XTT22 (*ShXTT22CAD*, 111).

As *Erianthus rufipilus* (a diploid species) is a more suitable reference genome for sugarcane than sorghum [33], *EruCAD* genes were named based on their chromosomal locations. Given the high genetic conservation of *CAD* genes and the polyploid complexity of sugarcane. In order to have a clear picture of the inter- and intraspecific homology between the genomes of *Erianthus rufipilus* and five sugarcane species. The study compared the homology of five sugarcane *CAD* gene family members with *Erianthus rufipilus CAD* gene family members and named them according to their homology with *EruCAD* genes, and assigned genes located on homologous chromosomes the same name with different chromosome letters, and genes that were not compared to each other continued to be named in sequential order.

Physicochemical characterization of *CAD* proteins across the six genomes revealed considerable diversity in their structural properties. The amino acid lengths ranged from 250 residues (*EruCAD9*) to 980 residues (*ShXTT22-CAD1-2B*), with corresponding molecular weights spanning from 26,983.79 Da (*EruCAD9*) to 107,725.11 Da (*ShXTT22-CAD1-2B*). The isoelectric points (pI) exhibited a broad range from 4.96 (*ShXTT22CAD2-2I1*) to 10.47 (*SoCAD3-2A2*), with a mean pI of 6.71. Notably, 223 *CAD* proteins (69.2% of the total) were classified as acidic proteins based on their pI values (Supplementary Table S2).

### 3.6. Evolutionary Analysis of the Sugarcane CAD Gene Family

**Figure 5:**
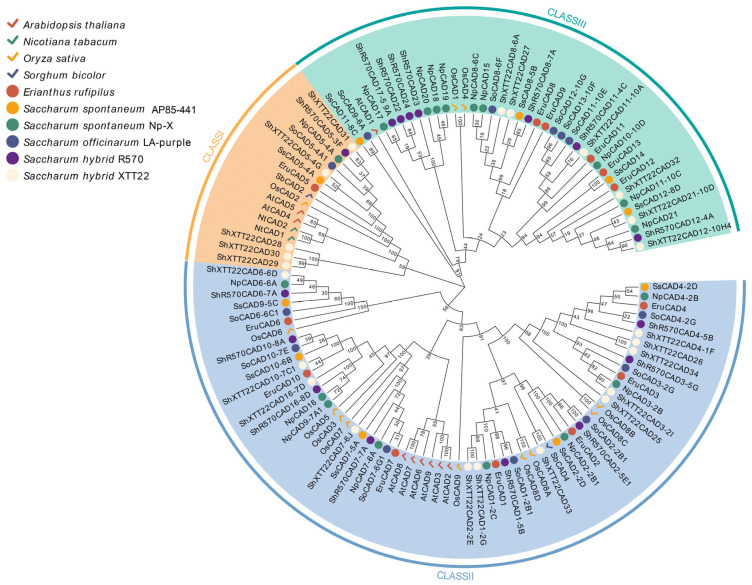
Neighbor-joining (NJ) phylogenetic tree of *CAD* genes from *Arabidopsis thaliana*, *Nicotiana tabacum*, *Oryza sativa*, *Sorghum bicolor*, *Erianthus rufipilus*, and five sugarcane varieties. Blue indicates Class III *CAD* genes, while red indicates genes from *Arabidopsis thaliana*, *Nicotiana tabacum*, *Oryza sativa*, and *Sorghum bicolor*, represented by checkmarks in distinct colors. Genes from *Erianthus rufipilus*, *S. spontaneum* AP85-441, *S. spontaneum* Np-X, *S. officinarum* LA-Purple, *S. hybrid* R570, and *S. hybrid* XTT22 are depicted as circles in varying colors.

To better evaluate the evolutionary relationships among *CAD* proteins in sugarcane, this study constructed a neighbor-joining (NJ) phylogenetic tree using 121 *CAD* genes (Genes with >90% sequence homology were grouped as orthologs.), including representative homologs from sugarcane (*SsCAD*, *NpCAD*, *SoCAD*, *ShR570CAD*, *ShXTT22CAD*) with *Arabidopsis thaliana* (*AtCAD*), *Nicotiana tabacum* (*NtCAD*), *Oryza sativa* (*OsCAD*), *Sorghum bicolor* (*SbCAD*), and *Erianthus rufipilus* (*EruCAD*). Following the established classification criteria for *CAD* gene families, the tree was divided into three classes, consistent with previous studies (Figure 5). Class I includes *AtCAD4* and *AtCAD5*, which are recognized as a teru *CAD* critical for lignin biosynthesis. Notably, all sugarcane *CAD5* homologs clustered within this group. Class II contains *AtCAD7* and *AtCAD8*, genes associated with plant stress resistance; the sugarcane *CAD* has the most members in this grouping, suggesting potential functional diversification in sugarcane. Class III, represented by *AtCAD1* (which lacks a direct role in lignin synthesis), requires further investigation to clarify its functional relevance in lignin metabolism. The sugarcane formed an independent evolutionary branch distinct from other Poaceae (the grass family) species, indicating significant lineage-specific expansion of the *CAD* gene family and divergent selection pressures during evolution.

**Figure 6:**
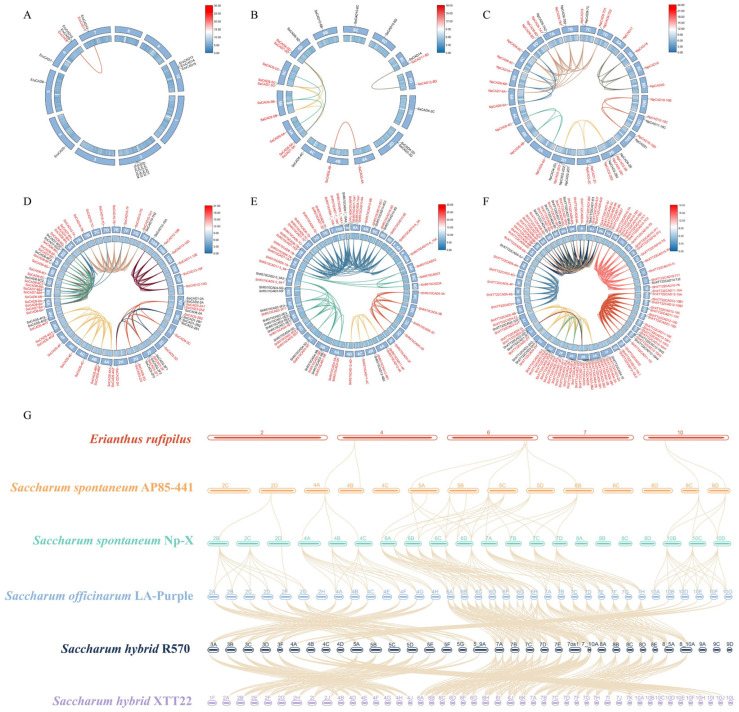
Intra- and inter-species covariance comparisons of six sugarcane. *CAD* genes involved in segmental duplication are highlighted in red. (**A**–**F**) represent interspecific synteny maps for *Erianthus rufipilus*, *S. spontaneum* AP85-441, *S. spontaneum* Np-X, *S. officinarum* LA-Purple, *S. hybrid* R570, and *S. hybrid* XTT22, respectively. The inner ring of each panel displays the gene density gradient (red to blue) across chromosomes, while the outer ring annotates chromosome labels. Colored lines connect syntenic sugarcane *CAD* gene pairs. (**G**) Illustrates interspecific synteny relationships among all six sugarcane complexes, with lines connecting syntenic *CAD* genes across species.

This study analyzed the chromosomal localisation of *CAD* genes (Supplementary Figure S2) and performed intra- and inter-species covariance comparisons for six sugarcane (Figure 6), and calculated Ka/Ks values for *CAD* genes of 96 representative sugarcane (Supplementary Table S3). By analyzing the intraspecific covariance of *CAD* genes in the six sugarcane varieties, this study found that there were a large number of gene fragment duplication events (covariate pairs of genes with the same gene connected by lines of the same color), and that these duplications occurred mainly between homologous chromosomes. Extensive covariant region connectivity reveals that a large number of homologous genes are shared between these chromosomes, and also reflects a high degree of conservation among species. As the ploidy of sugarcane chromosomes increased, the proportion of genes undergoing segmental duplications increased significantly, and the complexity of the covariate linkages also increased, suggesting that genomic relationships tended to be complex and revealed genomic rearrangements on specific chromosomes as well as cross-chromosomal gene exchange phenomena. Through a comparative multi-species genome analysis (Figure 6G), this study found a large number of conserved covariance regions between *Erianthus rufipilus* and the other five sugarcane species, reflecting their common ancestry. Hybrid sugarcane varieties (e.g., R570 and XTT22) exhibited remarkable genomic diversity, likely driven by extensive chromosomal recombination and structural variations during hybridization. Differential gene expression or copy number variations (gradient from red to blue in the inner rings of Figure 6A–F) were unevenly distributed across chromosomes, indicating that specific chromosomes may play key roles in environmental adaptation or functional evolution.

Ka/Ks analysis of 96 representative sugarcane *CAD* genes showed all values < 1; it is further suggested that these expanded gene families may have experienced different selective pressures, with most of them subject to purifying selection and relatively conserved functions. However, the *ShXT722CAD8-6A/ShR570CAD8-7A* pair exhibited a Ka/Ks ratio of 0.721, approaching 1, implying potential positive selection linked to adaptive evolution in sugarcane. These gene expansion events, mediated by tandem and segmental duplications, provide a critical genetic foundation for adaptive evolution in the *Saccharum* genus, enabling enhanced environmental resilience and functional diversification.

### 3.7. Conserved Motifs, Protein Domains, and Gene Structures of Sugarcane CAD Genes

**Figure 7:**
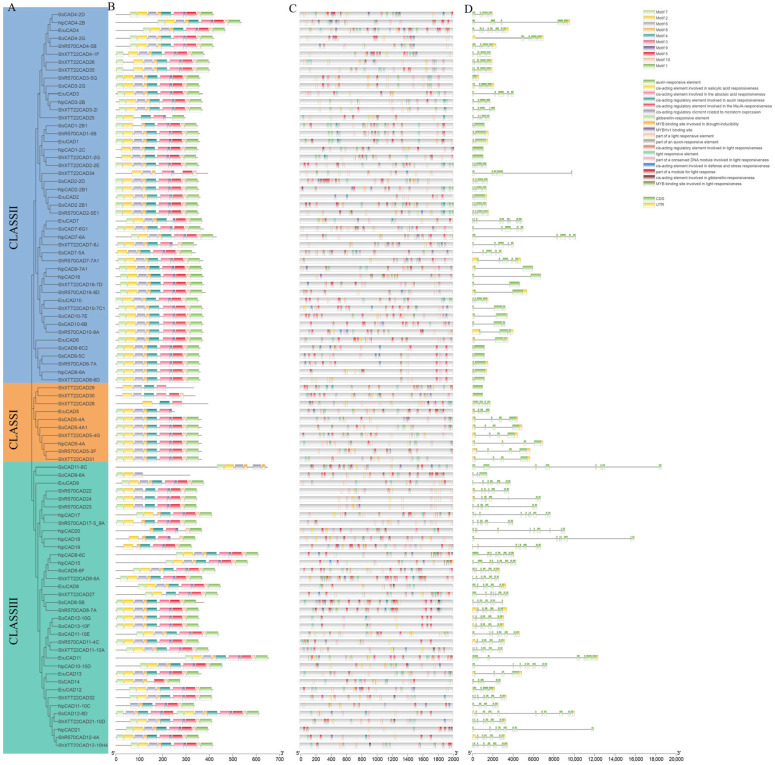
Phylogenetic relationships, conserved motifs, cis-acting elements, and gene structures of the sugarcane *CAD* gene family. (**A**) A neighbor-joining (NJ) phylogenetic tree was constructed using full-length protein sequences of the sugarcane complex in MEGA11, with 1000 bootstrap replicates to assess branch reliability. (**B**) Distribution of conserved motifs 1–10 across sugarcane *CAD* proteins, represented by colored boxes. (**C**) Cis-acting regulatory elements identified in promoter regions of sugarcane *CAD* genes. (**D**) Exon-intron organization of sugarcane *CAD* genes. Exons (yellow rectangles) and untranslated regions (UTRs, green rectangles) are interspersed by introns (black lines).

This study analyzed conserved motifs and protein domains of 96 selected sugarcane *CAD* gene family members using the MEME and Batch CD-Search online tools (Supplementary Figure S3), predicting 10 conserved motifs (Figure 7B). The number and distribution of motifs varied among different sugarcane *CAD* proteins. With the exception of *ShXTT22CAD28*, *ShXTT22CAD29*, and *SoCAD9-6A*, each member contained 6–10 motifs, with the majority harboring all 10 motifs, indicating high homology within the sugarcane *CAD* gene family. Most Class I members possessed all 10 motifs, and all sugarcane *CAD* proteins shared a conserved Motif 2. The N-terminal regions of these proteins were highly conserved, while the C-terminal regions exhibited relatively lower conservation. Most genes shared certain core motifs, suggesting these regions are critical and evolutionarily conserved across the gene family. Notably, Class II genes displayed the highest diversity in motif composition and quantity, whereas Class I and Class III genes had fewer motifs. Additionally, Class II genes exhibited more complex gene structures compared to the relatively simpler architectures of Class I and Class III genes.

These findings collectively highlight that sugarcane *CAD* gene family members share high homology but also display structural and functional divergence. Differences in motif composition and gene structure among subfamilies likely reflect distinct biological functions associated with each subfamily.

### 3.8. Cis-Acting Element Analysis

To explore the biological functions of sugarcane *CAD* genes, this study extracted 2000 bp upstream sequences of these genes as putative promoter regions and analyzed the cis-acting elements within these regions. The results revealed diverse cis-acting elements associated with plant hormone signaling, stress responses, developmental regulation, and abiotic stress adaptation (Supplementary Figure S4). Among the 96 sugarcane *CAD* genes analyzed, 76 contained light-responsive elements, 47 had auxin-responsive elements, 25 possessed gibberellin-responsive elements, and 14 carried elements linked to defense and stress responses. Additional elements identified included those responsive to salicylic acid, abscisic acid, methyl jasmonate (MeJA), drought induction, and meristem-specific expression. This study found that multiple *CAD* genes contain ABA response elements (ABREs), and the ABRE (ACGTG core sequence) of these genes exhibit high similarity to the confirmed ABI5 transcription factor binding sites in Arabidopsis *AtCAD1*. In terms of methyl jasmonate (MeJA) response, most *CAD* gene promoters contain a typical CGTCA-motif, and their expression patterns are similar to the JA regulatory mechanism of tobacco *NtCAD*, i.e., rapid induction of expression under mechanical injury or insect pest stress. These results collectively suggest that sugarcane *CAD* genes may respond to ABA and MeJA signals through conserved cis-regulatory elements, thereby regulating lignin biosynthesis to adapt to environmental stress [34,35].

The presence of varied cis-acting elements in the promoter regions suggests that sugarcane *CAD* genes participate in multiple biological processes and regulatory pathways. Most genes contained multiple elements related to light and stress responses, indicating their critical roles in environmental signal transduction. Notably, Class II genes exhibited significantly higher diversity in promoter elements compared to Class I and Class III, implying their regulation by more complex transcriptional networks.

### 3.9. Transcriptome Heatmap Analysis

**Figure 8:**
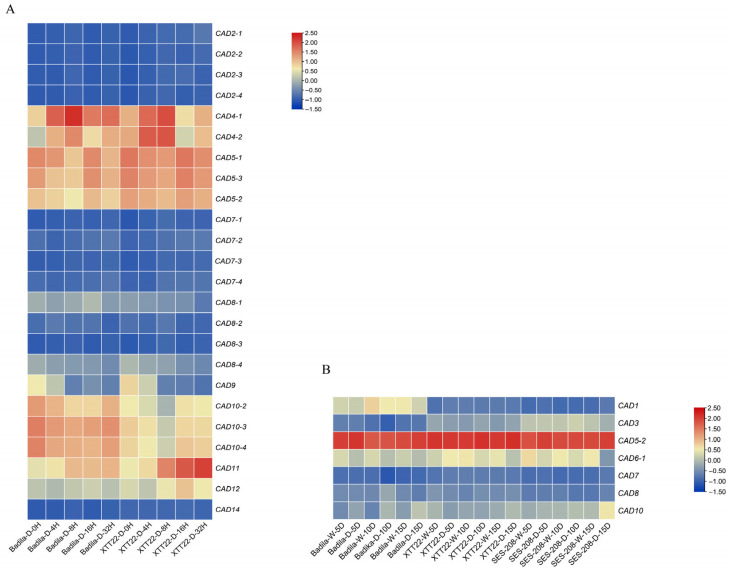
Differential expression heatmap of *CAD* genes under drought stress. (**A**) Leaf expression profiles of *CAD* genes in *Badila* and XTT22 sugarcane cultivars under drought treatments for 4, 8, 16, and 32 h, compared to well-watered controls (W). (**B**) Root expression patterns of *CAD* genes in Badila, XTT22, and SES-208 genotypes under drought stress applied for 5, 10, and 15 days, alongside well-watered conditions (W). Color gradients reflect Fragments Per Kilobase per Million mapped reads (FPKM) values, illustrating transcriptional dynamics in response to drought. Key genes with marked expression changes are highlighted, emphasizing their potential roles in drought adaptation across tissues and genotypes.

This study performed differential expression analysis of sugarcane *CAD* homologs using transcriptome data from *Saccharum officinarum* LA-Purple roots and *Saccharum spontaneum* AP85-441 leaves under drought stress (Figure 8). In roots, seven *CAD* genes showed distinct expression patterns between normal and drought conditions, with *CAD5-2* (Class I) displaying the highest expression across all time points, consistent with the pivotal role of Class I genes in lignin biosynthesis. In leaves, 24 *CAD* homologs exhibited differential expression in Badila and XTT22 after 4, 8, 16, and 32 h of drought treatment. Among these, *CAD4-1*, *CAD4-2*, *CAD5-1*, *CAD5-2*, *CAD5-3*, *CAD10-2*, *CAD10-3*, *CAD10-4*, *CAD11*, and *CAD12* showed significantly higher expression compared to other genes, suggesting their specialized roles in drought resistance. Notably, *CAD5-2* was highly expressed in both roots and leaves, highlighting its importance in sugarcane drought adaptation.

### 3.10. Drought-Induced Expression of CAD Genes in Sugarcane Roots

**Figure 9:**
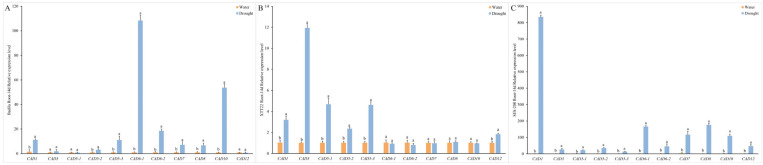
Quantitative real-time PCR (qRT-PCR) analysis of 11 *CAD* genes in roots under drought stress and control conditions at 14 days. (**A**) Badila Root-14d Relative expression level. (**B**) XTT22 Root-14d Relative expression level. (**C**) SES-208 Root-14d Relative expression level. The bar graphs display relative expression levels calculated using the 2^−ΔΔCT^ method, with data presented as mean ± standard deviation (SD). Error bars represent SD, and different lowercase letters above the bars indicate statistically significant differences compared to the control group (*p* < 0.05).

Through homology comparison of *CAD* genes across six sugarcane, this study selected 11 conserved *CAD* sequences present in all six genomes, hypothesizing their high homology and genetic stability (Figure 9). These 11 *CAD* genes were subjected to RT-qPCR analysis to assess their expression in roots of Badila, XTT22, and SES-208 under 14 days of drought stress. The tested genes included *CAD5-1*, *CAD5-2*, and *CAD5-3* (Class I); *CAD1*, *CAD3*, *CAD6-1*, *CAD6-2*, *CAD7*, and *CAD10* (Class II); and *CAD8* and *CAD12* (Class III).

The results showed that drought stress significantly upregulated eight *CAD* genes in Badila, six in XTT22, and all tested genes in SES-208. Notably, *CAD1*, *CAD5-2*, and *CAD5-3* exhibited marked expression increases across all three genotypes. *CAD1* reached its highest expression in SES-208, while *CAD5-2* showed the most pronounced upregulation in XTT22, consistent with transcriptome data highlighting its high expression in both drought-stressed roots and leaves. *CAD5-2* (Class I) is homologous to *AtCAD4/5* in *Arabidopsis*, and *CAD1* (Class II) corresponds to sugarcane *SAD* (sinapyl alcohol dehydrogenase). These findings suggest that *CAD* and *SAD* homologs positively regulate the accumulation of coniferyl alcohol and sinapyl alcohol, key intermediates in lignin biosynthesis, under drought stress.

### 3.11. Regulatory Mechanism of Lignin Accumulation Enhancing Drought Tolerance in Plants

**Figure 10:**
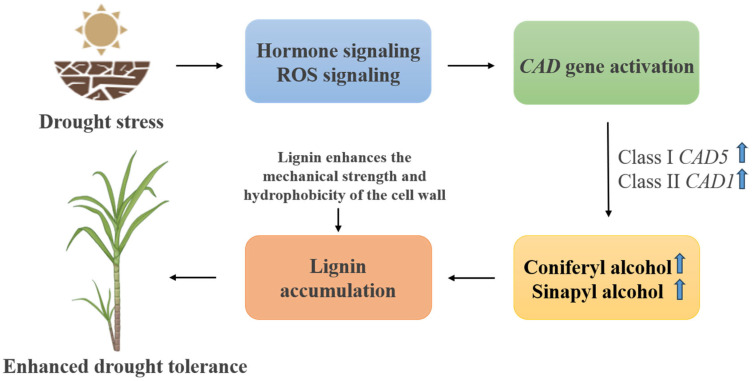
Regulatory mechanism of lignin accumulation enhancing drought tolerance in sugarcane.

Under drought stress, the expression levels of *CAD* and *SAD* genes were significantly upregulated, accompanied by increased accumulation of lignin monomers (coniferyl alcohol and sinapyl alcohol) and enhanced lignin deposition (Figure 10). Phenotypic observations revealed that drought-resistant accessions exhibited stronger cell wall rigidity, reduced leaf curling, and improved root vitality compared to sensitive varieties. These physiological changes were positively correlated with lignin content and *CAD/SAD* gene expression patterns.

## 4. Discussion

Lignin enhances plant drought tolerance as the second most abundant natural organic compound in plants after cellulose, serving both as a vital biomass energy source and a key regulatory metabolite under abiotic stress [36]. This study elucidates the critical role of lignin in sugarcane drought resistance by analyzing lignin content dynamics and related gene expression under drought stress. The results demonstrated that drought-tolerant sugarcane genotypes (e.g., XTT22 and SES-208) exhibited significant lignin accumulation, particularly in roots, under drought conditions, consistent with the protective role of lignin in drought resistance reported by Han et al. [37]. Notably, SES-208 showed a marked decline in leaf and root lignin content (*p* < 0.05) at 21 days of drought, likely due to metabolic suppression and lignin degradation under prolonged stress [38].

Coniferyl alcohol and sinapyl alcohol are pivotal monomers in lignin biosynthesis in sugarcane. Lignin polymers are derived from three precursors—p-coumaryl, coniferyl, and sinapyl alcohols—which form p-hydroxyphenyl (H), guaiacyl (G), and syringyl (S) lignin units, respectively [38]. In this study, XTT22 roots displayed significant increases in coniferyl and sinapyl alcohol levels under drought, confirming their essential role in enhancing sugarcane lignin biosynthesis. These findings align with Bang et al. [14], suggesting that lignin monomer accumulation improves water transport efficiency by strengthening vascular tissue rigidity.

Our investigation of six sugarcane hybrids focused on chromosomal localization, gene structure, conserved motifs, and synteny. qRT-PCR and transcriptome analyses further validated the positive role of sugarcane *CAD* genes in lignin synthesis and drought adaptation. This indicates that the *CAD* gene is closely related to normal plant growth and changes in plant lignin content, which is consistent with reports revealing that persimmon *WRKY* transcription factors (*DkWRKY8* and *DkWRKY10*) promote lignin accumulation in persimmon leaves by binding to the *DkCAD1* promoter [39]. To improve gene annotation accuracy, stringent criteria—including BLAST homology, CDD, HMMER, and conserved motif analyses—were applied. Protein properties, gene structures, and phylogenetic relationships revealed functional diversity: Class I genes, with complex exon-intron architectures, likely generate protein isoforms via alternative splicing for diverse biological roles, while Class III genes exhibit structural simplicity and functional conservation. Cis-regulatory element distribution highlighted Class II genes’ involvement in environmental stress responses.

Homologous evolution drives Class II expansion to enhance sugarcane XTT22 stress tolerance. Naming members of six sugarcane *CAD* gene families according to homology revealed that *CAD2* and *CAD10* had the most homologous genes, 36 each, and the vast majority of these genes clustered into Class II. It has been reported that members of the *CAD* gene family Class II are more homologous to *SAD*, that this subgroup is not responsible for lignin biosynthesis, that it is associated with plant resistance, and that frequent gene duplications and losses occur in members of this group [21]. Based on the phylogenetic tree analysis and homology identification results, it was found that the members of Class II were widely distributed in different branches, which might originate from the selective pressure of environmental adaptation and functional needs, and the Class II gene family had significant evolutionary diversity and complexity, which indicated that the gene family had experienced a complex process of amplification, loss and functional differentiation during the long term evolutionary process, and this result was in This result is consistent with previous studies [21]. Through phylogenetic tree analysis, 10 genes were identified in XTT22 that were not homologous to *CAD* genes of the other five sugarcane complexes, of which four clustered into Class I and four into Class II, which may also be the genetic basis for the better resistance of XTT22. Class I genes dominate lignin biosynthesis, exemplified by *EruCAD5* and its homologs across five sugarcane hybrids. Differential expression analysis revealed *CAD5-2* (Class I) as the most highly expressed gene across all tissues and stages. Metabolomic data showed drought-induced accumulation of coniferyl and sinapyl alcohols, while qRT-PCR confirmed coordinated expression patterns of *CAD1* and *CAD5* with these metabolites. This study proposes that *CAD5* catalyzes coniferyl alcohol synthesis, while *CAD1* drives sinapyl alcohol production, collectively boosting lignin content and drought resilience.

In the phylogenetic tree, members in Class I are genes that play major functions in lignin synthesis in sugarcane. This study found that *EruCAD5* and its homologous genes in five sugarcane species were clustered into Class I. This study analyzed *CAD* for differential expression in roots and leaves and found that *CAD5-2* (Class I) had the highest expression at all times. In addition, through metabolomics analysis, this study found that drought stress increased the content of small molecules in the lignin metabolic pathway, coniferyl alcohol and erucic alcohol, and our qRT-PCR of 11 *CAD* sequences revealed that the expression patterns of the *CAD* family genes, *CAD1* and *CAD5*, were consistent with the trends of these two small molecules. Combined with previous studies, this study speculated that the sugarcane complex *CAD5* is a key gene for catalyzing the synthesis of Coniferyl alcohol and *CAD1* is a key gene for catalyzing the synthesis of Sinapyl alcohol, and that *CAD5* plays an important role in the synthesis of lignin in sugarcane [40]. *CAD5* and *CAD1* catalyzed the synthesis of Coniferyl alcohol from Sinapyl alcohol to increase the lignin content of sugarcane, which in turn improved drought tolerance. These results provide a basis for further research on the role of the sugarcane *CADs* in the growth and development of sugarcane.

Gene duplication may be an important reason for the expansion and high degree of homology of this gene family in sugarcane. This study found that a large number of tandem duplication and segmental duplication events occurred in the sugarcane *CAD* family, and the percentage of interchromosomal segmental duplications increased with the increase in chromosome ploidy in sugarcane. Despite the high distribution of *CAD* genes in sugarcane, their conserved motifs and gene structures were basically similar, which also indicated a high degree of homology of *CAD* genes in genetic evolution. The interaction of purifying selection and positive selection mediated by tandem and segmental repeats has shaped the functional landscape of key gene families, both maintaining functional conservation and promoting functional innovation. In the context of drought tolerance, amplification of gene families is critical for improving stress tolerance, and these duplications not only amplify the expression of drought-responsive genes, but also enable plants to become more adaptive to their environment. Prior studies, such as *Populus* PuC3H35-mediated root lignification and antioxidant regulation [41], support our findings that lignin biosynthesis strengthens drought resistance via structural reinforcement and vascular development. By analyzing lignin dynamics and *CAD* expression in Badila, XTT22, and SES-208 under drought, this study deciphered the interplay between lignin metabolism and drought adaptation in sugarcane.

The observed phenomenon in the Badila variety, where *CAD* genes were significantly upregulated under stress conditions without a corresponding change in total lignin content, may reflect the complexity of lignin metabolic regulation. Although the overall lignin content remained stable, the upregulation of *CAD* genes could potentially modify cell wall properties by altering lignin monomer composition (e.g., S/G ratio), a mechanism previously documented in Arabidopsis *CAD* mutants [42]. Notably, the upregulated *CAD* genes in Badila predominantly belong to Class II/III, which phylogenetically appear more involved in plant stress responses than lignin biosynthesis, suggesting their potential role in alternative pathways such as localized cell wall reinforcement or stress-related metabolism. This interpretation is supported by comparative data from two other varieties (SES-208 and XTT22): SES-208 exhibited coordinated increases in both *CAD* expression and lignin content, while XTT22 displayed an intermediate phenotype. These varietal differences indicate that *CAD*-mediated regulation of lignin biosynthesis may follow a dose-dependent threshold effect, with Badila potentially representing a critical transitional state within this regulatory spectrum. These findings provide new insights into the sophisticated regulatory mechanisms governing lignin synthesis.

This study investigated the changes in lignin content of sugarcane and the expression pattern of its *CAD* genes under drought stress in Badila, XTT22, and SES-208 to explore the relationship between drought stress and changes in lignin content and *CAD* gene expression in sugarcane. This study detected the changes in lignin content in sugarcane under drought conditions, the changes in the content of small molecules of lignin metabolites, and the effects of *CAD* genes on lignin to gain a deeper understanding of drought-resistant mechanisms in sugarcane.

This study clarified that the sugarcane *CAD* gene family has a significant role in synthesizing lignin and improving drought resistance in sugarcane by examining the expression patterns of various compounds in lignin and its metabolic pathways, as well as related genes [41]. This study provides potential targets for analyzing the drought resistance mechanism and mining resistance genes in sugarcane, and opens up a new way for analyzing the genetic basis of resistance formation in complex polyploid crops. The results not only provide a new direction for drought resistance enhancement in sugarcane but also provide an important reference value for the application of lignin and its related genes in plant resistance research. Future studies can further explore the functional differentiation of the *CAD* gene family and its expression regulation network under different environmental stresses, with a view to providing more scientific basis for drought tolerance breeding in sugarcane and other crops.

## Data Availability

The original contributions presented in this study are included in the article/Supplementary Material. Further inquiries can be directed to the corresponding authors.

## Supplementary Materials

The following supporting information can be downloaded at https://www.mdpi.com/article/10.3390/plants14172735/s1. Figure S1: Thirteen Metabolite Content Chart, Figure S2: Chromosomal localisation of the *CAD* gene family, Figure S3: Identification of conserved structural domains in *CAD* gene family proteins, Figure S4: Cis-acting regulatory elements identified in promoter regions of sugarcane *CAD* genes.,Table S1: qRT-PCR primers, Table S2: physicochemical properties of proteins in *Saccharum* spp., Table S3: Ka/Ks Analysis of CAD Genes in *Saccharum* spp.
