# Supplementary material for: Lignin Biosynthesis Driven by CAD Genes Underpins Drought Tolerance in Sugarcane: Genomic Insights for Crop Improvement
Source: Plants (Basel). 2025 Sep 2;14(17):2735. doi: 10.3390/plants14172735 (PMC12430416; doi:10.3390/plants14172735)
Supplement: Supplementary file 1 [file plants-14-02735-s001.zip › Figure S1/Figure S1 describe.pdf]

Figure S1: (A) Separate plots showing the content of 13 metabolites involved in lignin synthesis in leaves and roots at 14 days under drought and control conditions. (B) Combined plot showing the content of 13 metabolites involved in lignin synthesis in leaves and roots at 14 days under drought and control conditions. (C) Clustered heatmap of the 13 metabolites involved in lignin synthesis in leaves and roots at 14 days post-drought and control. R-Treat-14d: roots under drought treatment for 14 days; R-CK: roots under normal growth for 14 days; L-Treat-14d: leaves under drought treatment for 14 days; L-CK: leaves under normal growth for 14 days.

Index	**Compounds**	**Class**
pme3443	**Sinapinaldehyde**	**Phenylpropanoids**
pme3456	**p-Coumaraldehyde**	**Phenylpropanoids**
pme0021	**L-Phenylalanine**	**Amino acid and derivatives**
pme1695	**Sinapic acid**	**Phenylpropanoids**
pme1637	**Coniferyl alcohol**	**Phenylpropanoids**
pme1436	**p-Coumaric acid**	**Phenylpropanoids**
pme0303	**Caffeate**	**Phenylpropanoids**
pme0305	**Ferulic acid**	**Phenylpropanoids**
pme3123	**Sinapyl alcohol**	**Phenylpropanoids**
pme3305	**p-Coumaryl alcohol**	**Phenylpropanoids**
pmf0284	**4-Hydroxy-3-methoxycinnamaldehyde**	**Phenylpropanoids**
pmf0591	**Cinnamic acid**	**Phenylpropanoids**
pmb0142	**Caffeyl aldehyde**	**Phenylpropanoids**
