# Supplementary material for: Lignin Biosynthesis Driven by CAD Genes Underpins Drought Tolerance in Sugarcane: Genomic Insights for Crop Improvement
Source: Plants (Basel). 2025 Sep 2;14(17):2735. doi: 10.3390/plants14172735 (PMC12430416; doi:10.3390/plants14172735)
Supplement: Supplementary file 1 [file plants-14-02735-s001.zip › Supplementary Table.pdf]

Table S1 qRT-PCR primers

Primer number	Primer sequences（5’-3’）
CAD1-2F-F	TGGTCACCTCTCCCCTTACC
CAD1-2F-R	TCTTGATGACGTGGAGGTCG
CAD3-2F-F	GGAAACGCCATGTACCCTGT
CAD3-2F-R	CGCAGTAGTTCTCGTTCCCC
CAD5-4A2-F	CCGACTCGCTGGACTACATC
CAD5-4A2-R	CATCACCATTGGGGACACGA
CAD5-4C-F	TGATCAGCTCGTCGTCCAAG
CAD5-4C-R	GGCACCGTGTCGATGATGTA
CAD5-4G2-F	GTGATCAGCTCGTCGTCCAA
CAD5-4G2-R	GATGTAGTCCAGCGAGTCGG
CAD8-6B-F	CTCGACCATTGTCTCGTGCT
CAD8-6B-R	ACTGCTCTGCGGTTGAACTT
CAD6-6C1-F	CGACCTGCACTTCATCCAGA
CAD6-6C1-R	CGCAGTAGTTCTCCTCGGAC
CAD6-6D-F	TCGTTCCCGCTCATCTTTGG
CAD6-6D-R	CGATGTCGCAGGTGATGTTG
CAD7-6G1-F	AGGGACACTGGAGATGACGA
CAD7-6G1-R	GGGTAGTTGGCGTTCTTCCA
CAD10-7F-F	CACCTACAACTCCGTGGACC
CAD10-7F-R	GTTCAGGCCGTGGTACTTCA
CAD12-10A-F	CCGATGGCTATCCTTTGGCA
CAD12-10A-R	CACCAAGTCCAATGACCCCA
GADPH-F	CACGGCCACTGGAAGCA
GADPH-R	TCCTCAGGGTTCCTGATGCC

Table S2 physicochemical properties of proteins in *Saccharum* spp.

Sequence ID	Number of Amino Acid	Molecular Weight	Theoretical pI	Instability Index	Aliphatic Index	Grand Average of Hydropathicity
EruCAD1	358	37379.75	5.56	27.19	89.22	0.146
EruCAD2	354	37059.46	5.34	27.57	95.48	0.229
EruCAD3	653	67577.04	8.99	34.88	67.23	-0.379
EruCAD4	414	43371.81	6.91	30.12	89.18	0.105
EruCAD5	365	38657.49	5.94	24.15	94.47	0.132
EruCAD6	360	39125.03	7.17	32.86	89	-0.022
EruCAD7	370	39703.64	6.23	34.22	86.46	-0.045
EruCAD8	467	50391.26	8.49	36.2	77.86	-0.172
EruCAD9	250	26983.79	8.28	25.98	81.4	-0.067
EruCAD10	372	39508.63	7.15	26.91	82.58	0.004
EruCAD11	369	40021.81	6.79	28.42	87.43	-0.016
EruCAD12	447	48267.78	7.25	30.44	79.78	-0.184
EruCAD13	376	40847.74	6.39	24.47	86.28	-0.022
SsCAD2-2D	354	37166.66	5.47	27.04	95.17	0.226
SsCAD4-2C	418	43895.43	6.89	30.6	89.26	0.082
SsCAD4-2D	418	43876.34	6.92	31.62	88.56	0.07
SsCAD5-4A	365	38627.4	5.94	23.66	94.22	0.125
SsCAD5-4B	365	38627.47	5.94	23.13	94.49	0.139
SsCAD5-4C	365	38603.48	5.88	23.13	95.56	0.158
SsCAD7-5A	339	36360.79	5.78	29.91	84.84	-0.024
SsCAD8-5C	312	33488.39	5.39	31	84.65	-0.043
SsCAD8-5D	383	41401.45	5.97	28.25	83.99	-0.093
SsCAD8-5A	344	37553.86	6.82	29.66	82.7	-0.117
SsCAD8-5B	377	41012.59	6.49	32.4	81.41	-0.132
SsCAD7-5C	431	47544.03	7.17	32.36	76.82	-0.266
SsCAD7-5D	418	45493.97	6.88	36.29	84.14	-0.099
SsCAD9-5A	363	39321.14	6.63	33.11	90.14	0.001
SsCAD9-5B	360	39039.84	6.86	32.56	88.47	-0.02
SsCAD9-5C	360	39028.81	6.63	32.22	88.44	-0.016
SsCAD9-5D	360	39053.91	6.86	32.53	89	-0.012
SsCAD10-6B	373	39517.55	7.17	28.25	83.67	-0.013
SsCAD10-6C	375	39590.68	7.61	24.77	84.03	0.009
SsCAD10-6D	373	39551.64	7.17	26.76	83.94	0.005
SsCAD11-8C	648	71047.57	6.07	46.14	65.86	-0.46
SsCAD12-8D	612	66025.35	6.41	25.32	84.85	-0.04
SsCAD14	275	30165.18	6.45	27.14	83.56	-0.14
NpCAD1-2C	352	36912.49	6.14	31.34	91.25	0.19
NpCAD2-2B1	354	37216.67	5.46	28.14	95.48	0.209
NpCAD2-2B2	354	37216.67	5.46	28.14	95.48	0.209
NpCAD2-2B3	354	37147.58	5.35	30	93.56	0.199
NpCAD2-2D1	473	49717.75	5.05	28.17	97.82	0.161

NpCAD2-2D2	354	37161.61	5.34	29.6	94.1	0.205
NpCAD2-2D3	354	37161.61	5.34	29.6	94.1	0.205
NpCAD3-2B	370	38786.56	6.12	25.34	91.62	0.112
NpCAD3-2C	359	37469.08	6.26	24.26	92.26	0.146
NpCAD4-2B	536	56458.75	8.54	37.06	86.25	-0.038
NpCAD4-2D	418	43829.28	6.73	31.22	88.56	0.075
NpCAD5-4A	365	38655.52	5.94	23.13	95.01	0.145
NpCAD5-4B	365	38671.52	5.94	23.13	94.74	0.138
NpCAD5-4C	365	38655.52	5.94	23.13	95.01	0.145
NpCAD6-6A	360	39035.84	6.86	32.77	89.56	-0.015
NpCAD6-6B	360	39127.95	6.86	32.22	88.44	-0.028
NpCAD6-6C	360	39051.88	6.86	31.55	90.64	0
NpCAD6-6D	677	72955.58	8.43	53.15	75.13	-0.252
NpCAD7-6A	429	46221.75	6.76	44.92	79.58	-0.198
NpCAD8-6C	609	65311.04	8.35	48.41	73.83	-0.214
NpCAD8-6D	429	46168.46	6.82	30.81	80.42	-0.114
NpCAD9-7A1	369	39025.04	8.02	26.95	85.12	0.025
NpCAD9-7A2	374	39476.58	8.03	26.42	84.52	0.028
NpCAD9-7B1	376	39597.66	7.6	27.33	84.87	0.044
NpCAD9-7B2	374	39610.72	7.61	25.91	83.21	-0.002
NpCAD9-7C	373	39521.56	7.17	26.76	83.91	-0.008
NpCAD9-7D1	297	31618.71	8.39	26.37	85.96	0.051
NpCAD9-7D2	374	39603.69	8.03	27.4	84.25	0.004
NpCAD10-10B	396	42823.05	7.12	28.3	88.86	-0.012
NpCAD10-10C	396	42910.94	6.82	30	85.4	-0.063
NpCAD10-10D	455	48670.27	8.26	32.19	81.45	-0.134
NpCAD11-10C	335	36201.43	6.17	21.77	90.72	0.025
NpCAD15	564	60382.41	8.83	45.26	75.04	-0.213
NpCAD16	374	39509.59	8.02	26.53	85.56	0.04
NpCAD17	411	44664	8.8	36.08	78.1	-0.25
NpCAD18	340	36668.3	8.09	38.88	91.97	0.066
NpCAD19	324	35232.79	6.78	27.06	94.41	0.134
NpCAD20	368	39054.93	8.47	37.1	83.72	0.036
NpCAD21	395	42879.93	7.21	29.5	83.85	-0.114
SoCAD1-2A	348	36564.16	6.3	29.13	93.71	0.226
SoCAD1-2B1	350	36636.95	5.99	31.09	87.09	0.126
SoCAD1-2B2	343	36089.52	5.85	34.21	91.11	0.187
SoCAD1-2F	358	37518.98	5.87	30.43	88.1	0.145
SoCAD2-2A	354	37060.44	5.34	26.6	95.76	0.231
SoCAD2-2B1	354	37059.46	5.34	27.57	95.48	0.229
SoCAD2-2B2	354	37059.46	5.34	27.57	95.48	0.229
SoCAD2-2D	354	37138.6	5.47	28.28	94.63	0.214
SoCAD2-2F1	354	37138.6	5.47	28.28	94.63	0.214
SoCAD2-2F2	354	37079.44	5.34	27.33	95.2	0.224

SoCAD2-2H	354	37196.64	5.36	28.1	94.63	0.205
SoCAD3-2A1	359	37477.01	5.99	22.31	93.04	0.162
SoCAD3-2A2	603	63344.72	10.47	47.18	79.95	-0.183
SoCAD3-2F1	429	45095.7	7.22	30.44	89.04	0.02
SoCAD3-2F2	418	43980.51	6.91	31.43	88.09	0.063
SoCAD3-2G	359	37417.9	5.99	24.26	91.17	0.135
SoCAD4-2A	418	43964.45	6.92	31.86	88.09	0.056
SoCAD4-2C	514	53935.07	8.65	37.7	89.18	0.033
SoCAD4-2G	418	43976.5	6.92	31.72	88.78	0.063
SoCAD5-4A1	365	38669.59	6.06	23.01	95.01	0.144
SoCAD5-4A2	365	38669.59	6.06	23.01	95.01	0.144
SoCAD5-4B1	365	38669.59	6.06	23.01	95.01	0.144
SoCAD5-4B2	365	38669.55	5.94	23.66	95.29	0.151
SoCAD5-4C	365	38655.52	5.94	23.13	95.01	0.145
SoCAD5-4E	365	38669.59	6.06	23.01	95.01	0.144
SoCAD5-4F	365	38669.55	5.94	23.66	95.29	0.151
SoCAD5-4G1	365	38669.59	6.06	23.01	95.01	0.144
SoCAD5-4G2	311	32990.07	5.51	23.51	96.14	0.219
SoCAD5-4H1	365	38669.59	6.06	23.01	95.01	0.144
SoCAD5-4H2	365	38669.59	6.06	23.01	95.01	0.144
SoCAD5-4H3	365	38669.59	6.06	23.01	95.01	0.144
SoCAD6-6A	360	39129.92	6.86	31.53	87.64	-0.036
SoCAD6-6B	360	39029.76	6.63	31.35	87.36	-0.039
SoCAD6-6C2	360	39087.88	6.86	31.11	87.36	-0.041
SoCAD6-6C1	360	39087.88	6.86	31.11	87.36	-0.041
SoCAD6-6D	360	39074.94	6.63	31.64	88.97	0
SoCAD6-6F1	360	39087.88	6.86	31.11	87.36	-0.041
SoCAD6-6F2	360	39087.88	6.86	31.11	87.36	-0.041
SoCAD6-6G	360	39087.88	6.86	31.11	87.36	-0.041
SoCAD6-6H	360	39087.88	6.86	31.11	87.36	-0.041
SoCAD7-6A	382	40857.03	6.91	36.83	85.03	-0.035
SoCAD7-6B1	382	40838.93	6.43	34.89	85.03	-0.031
SoCAD7-6B2	370	39542.46	6.23	35.34	86.19	-0.028
SoCAD7-6F	375	40065.15	6.36	33.75	88.16	0
SoCAD7-6G2	375	40065.15	6.36	33.75	88.16	0
SoCAD7-6G1	483	52008.44	8.33	42.73	79.79	-0.202
SoCAD7-6H	361	38789.77	6.63	32.86	84.79	-0.005
SoCAD7-6H	370	39526.46	6.23	34.3	86.46	-0.021
SoCAD8-6A	491	52784.91	7.88	39.42	80.98	-0.165
SoCAD8-6B	729	78900.22	8.63	46.49	76.01	-0.341
SoCAD8-6C	387	41959.86	6.59	29.82	82.3	-0.074
SoCAD8-6D	759	82601.72	7.77	40.03	81.7	-0.194
SoCAD8-6F	425	46616.26	7.83	33.96	80	-0.187
SoCAD8-6G	425	46604.25	7.83	33.97	80.47	-0.178

SoCAD9-6A	315	33192.03	9.09	52.09	60.16	-0.524
SoCAD10-7A	373	39549.63	7.61	28.58	84.45	0.004
SoCAD10-7B	374	39605.74	7.17	27.89	85.27	0.024
SoCAD10-7C	367	38901.92	7.6	25.61	85.56	0.02
SoCAD10-7D	367	38972.02	7.6	26.7	86.35	0.031
SoCAD10-7E	374	39605.74	7.17	27.89	85.27	0.024
SoCAD10-7F	339	36157.75	6.25	24.61	87.4	0.051
SoCAD10-7G	374	39633.75	7.17	28.64	85.27	0.022
SoCAD10-7H1	374	39605.74	7.17	27.89	85.27	0.024
SoCAD10-7H2	325	34336.74	8.04	29.23	84.62	0.051
SoCAD11-10E	439	47427.21	8.73	26.54	83.03	-0.164
SoCAD12-10A	408	44228.39	7.59	35.5	80.25	-0.156
SoCAD12-10A	407	44238.43	8.55	35.48	80.2	-0.186
SoCAD12-10G	354	38521.15	6.55	23.73	87.26	-0.034
SoCAD13-10D	408	44309.51	8.55	34.8	80	-0.187
SoCAD13-10F	354	38521.15	6.55	23.73	87.26	-0.034
ShR570CAD1-5B	358	37518.98	5.87	30.43	88.1	0.145
ShR570CAD1-5E1	350	36636.95	5.99	31.09	87.09	0.126
ShR570CAD1-5E2	350	36636.95	5.99	31.09	87.09	0.126
ShR570CAD2-5A	354	37089.48	5.34	27.33	95.2	0.221

Table S3 Ka/Ks Analysis of CAD Genes in *Saccharum* spp.

Gene Name	Gene ID	Gene Name	Gene ID	Ka	Ks	Ka/Ks
SoCAD11-10E	Soffic.10G0006520-4E.t1	NpCAD10-10D	Npp.10D007180.1.t1	0.053669	0.095466	0.562178
SoCAD13-10F	Soffic.10G0004900-2F.t1	NpCAD10-10D	Npp.10D007180.1.t1	0.017503	0.066136	0.264647
SoCAD12-10G	LAp.10G0006340.t1	NpCAD10-10D	Npp.10D007180.1.t1	0.017503	0.066136	0.264647
ShR570CAD12-4B1	SoffiXsponR570.04Bg072900.1	SoCAD11-10E	Soffic.10G0006520-4E.t1	0.013718	0.032339	0.424177
ShR570CAD12-4B1	SoffiXsponR570.04Bg072900.1	SoCAD13-10F	Soffic.10G0004900-2F.t1	0.011207	0.036457	0.307406
ShR570CAD12-4B1	SoffiXsponR570.04Bg072900.1	SoCAD12-10G	LAp.10G0006340.t1	0.011207	0.036457	0.307406
ShR570CAD11-4C	SoffiXsponR570.04Cg077100.1	SoCAD11-10E	Soffic.10G0006520-4E.t1	0.00995	0.020055	0.496163
ShR570CAD11-4C	SoffiXsponR570.04Cg077100.1	SoCAD13-10F	Soffic.10G0004900-2F.t1	0.007452	0.024115	0.309022
ShR570CAD11-4C	SoffiXsponR570.04Cg077100.1	SoCAD12-10G	LAp.10G0006340.t1	0.007452	0.024115	0.309022
ShR570CAD10-8A	SoffiXsponR570.08Ag083700.1	SoCAD10-7E	LAp.07E0006080.t1	0.007139	0.037344	0.191158
ShR570CAD16-8D	SoffiXsponR570.08Dg050500.1	SoCAD10-7E	LAp.07E0006080.t1	0.041885	0.117156	0.357516
ShXTT22CAD11-10A	ROC_gene074920.t1	ShR570CAD12-4B1	SoffiXsponR570.04Bg072900.1	0.006202	0.028258	0.219466
ShXTT22CAD11-10A	ROC_gene074920.t1	ShR570CAD11-4C	SoffiXsponR570.04Cg077100.1	0.002474	0	\
ShXTT22CAD32	ROC_gene212672.t1	ShR570CAD12-4B1	SoffiXsponR570.04Bg072900.1	0.036732	0.10142	0.362174
ShXTT22CAD32	ROC_gene212672.t1	ShR570CAD11-4C	SoffiXsponR570.04Cg077100.1	0.038027	0.096943	0.392261
ShXTT22CAD31	ROC_gene207359.t1	ShR570CAD12-4B1	SoffiXsponR570.03Fg077900.1	0.001228	0.014415	0.085207
ShXTT22CAD5-4G	ROC_gene028081.t1	ShR570CAD12-4B1	SoffiXsponR570.03Fg077900.1	0.001228	0.007173	0.171237
ShXTT22CAD8-6A	ROC_gene084545.t1	ShR570CAD8-7A	SoffiXsponR570.07Ag205900.1	0.008684	0.012036	0.721485
ShXTT22CAD6-6D	ROC_gene106182.t1	ShR570CAD6-7A	SoffiXsponR570.07Ag016300.1	0.003701	0.026653	0.138848
ShXTT22CAD7-6J	ROC_gene108580.t1	ShR570CAD7-7A1	SoffiXsponR570.07Ag195500.1	0.016648	0.041376	0.402358
ShXTT22CAD10-7C1	ROC_gene138563.t1	ShR570CAD10-8A	SoffiXsponR570.08Ag083700.1	0.009552	0.04139	0.23079
ShXTT22CAD10-7C1	ROC_gene138563.t1	ShR570CAD16-8D	SoffiXsponR570.08Dg050500.1	0.038808	0.12067	0.321605

ShXTT22CAD16-7D	ROC_gene190898.t1	ShR570CAD16-8D	SoffiXsponR570.08Dg050500.1	0.012834	0.03606	0.355903
