# Supplementary figures and images for: Lignin Biosynthesis Driven by CAD Genes Underpins Drought Tolerance in Sugarcane: Genomic Insights for Crop Improvement
Source: Plants (Basel). 2025 Sep 2;14(17):2735. doi: 10.3390/plants14172735 (PMC12430416; doi:10.3390/plants14172735)

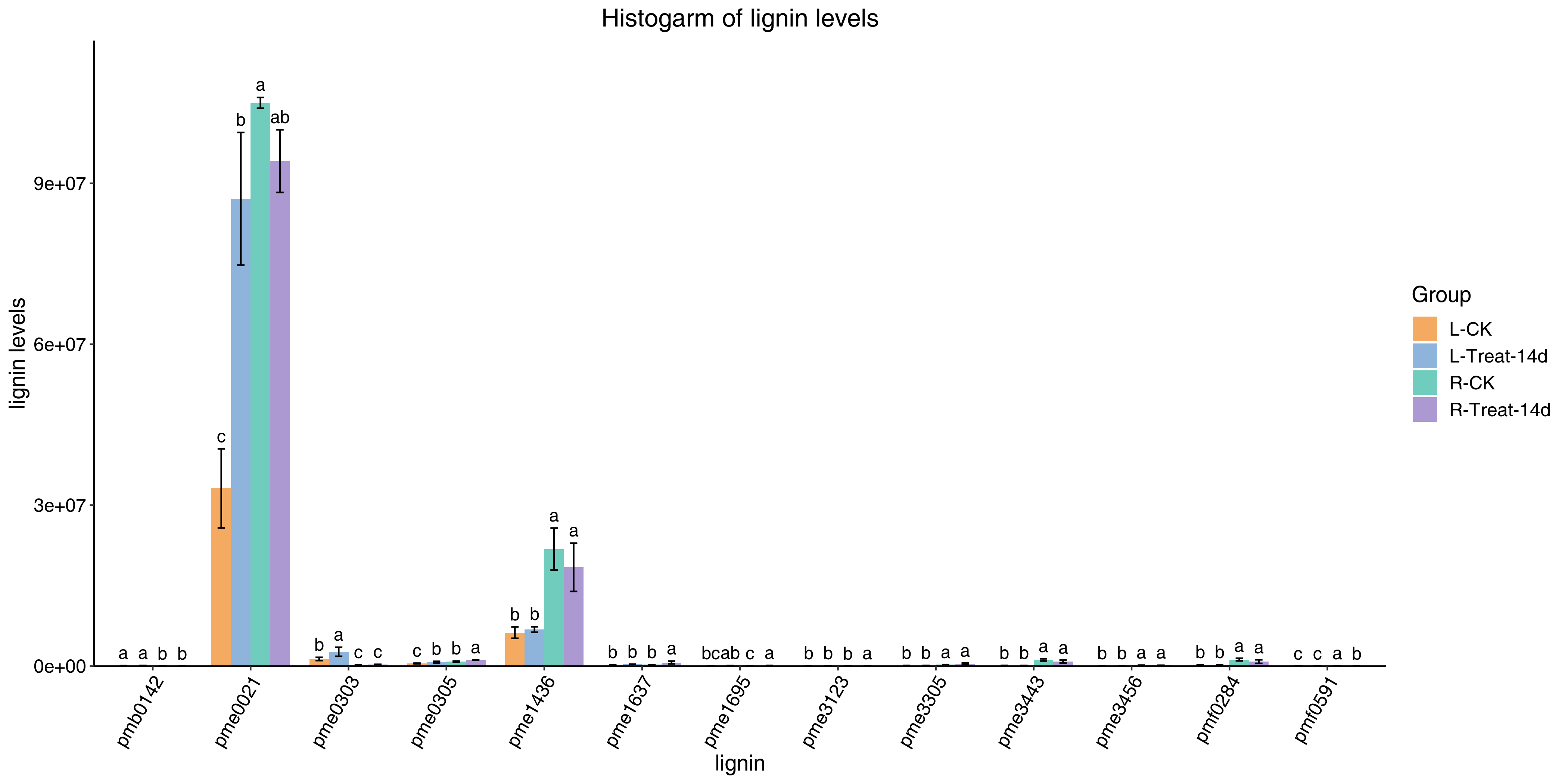

Supplement: Supplementary file 1 [file plants-14-02735-s001.zip › Figure S1/Figure_S1B .png]

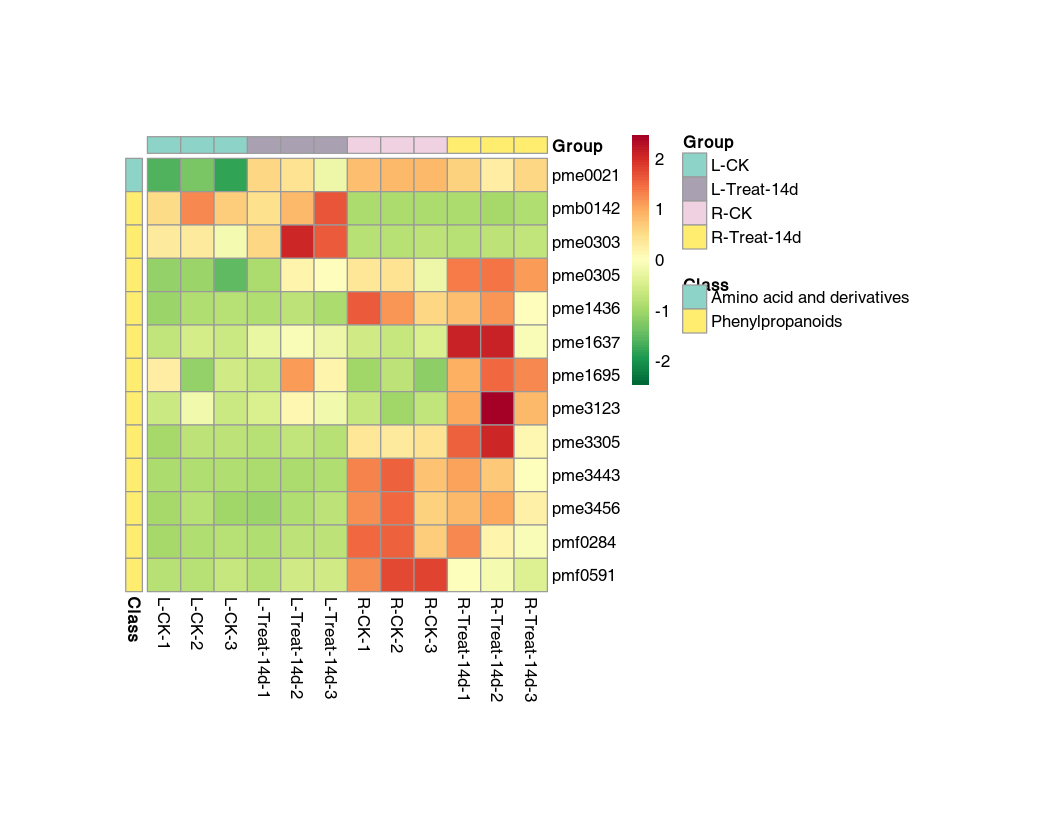

Supplement: Supplementary file 1 [file plants-14-02735-s001.zip › Figure S1/Figure_S1C.png]

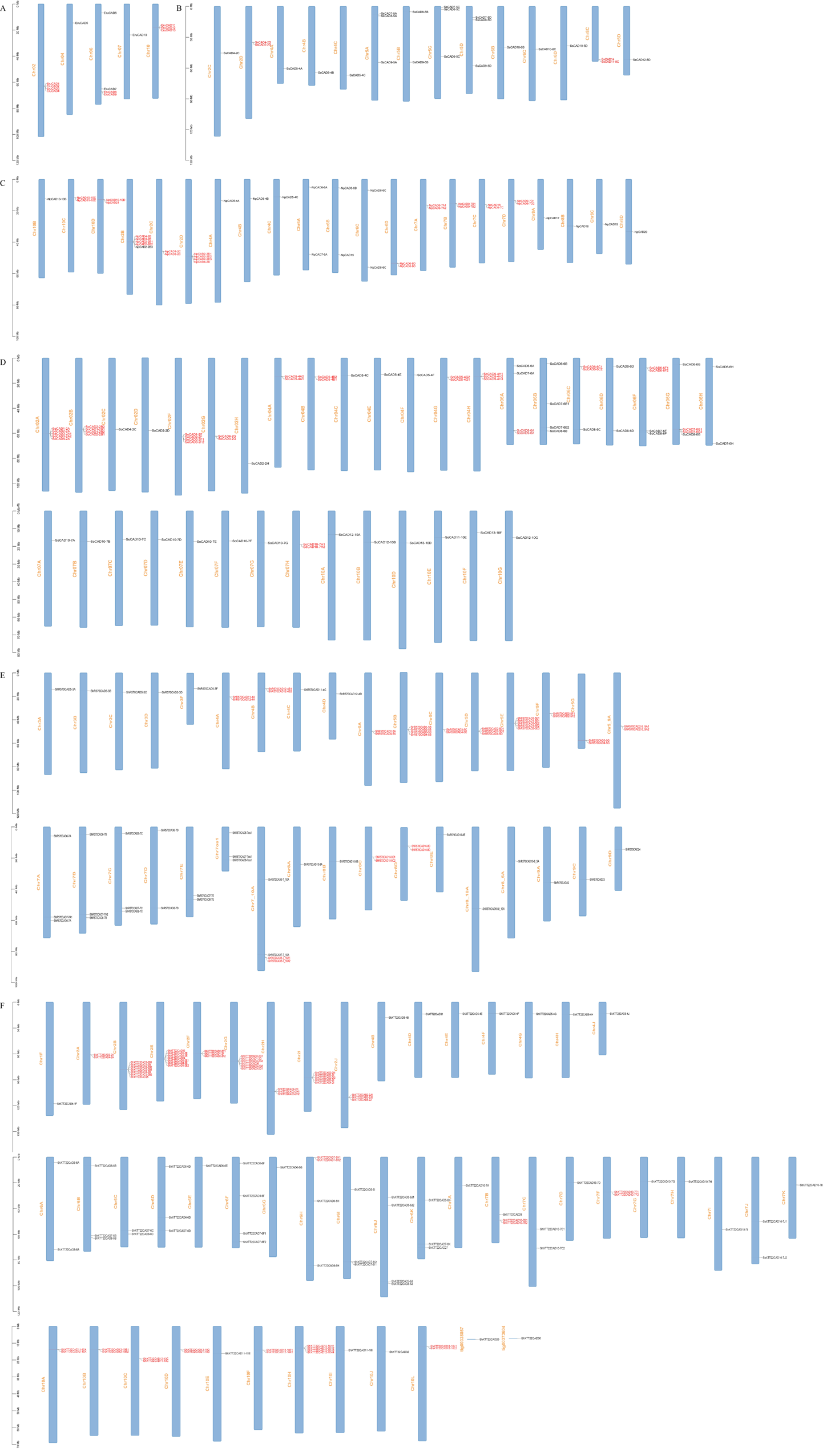

Supplement: Supplementary file 1 [file plants-14-02735-s001.zip › Figure S2.jpg]

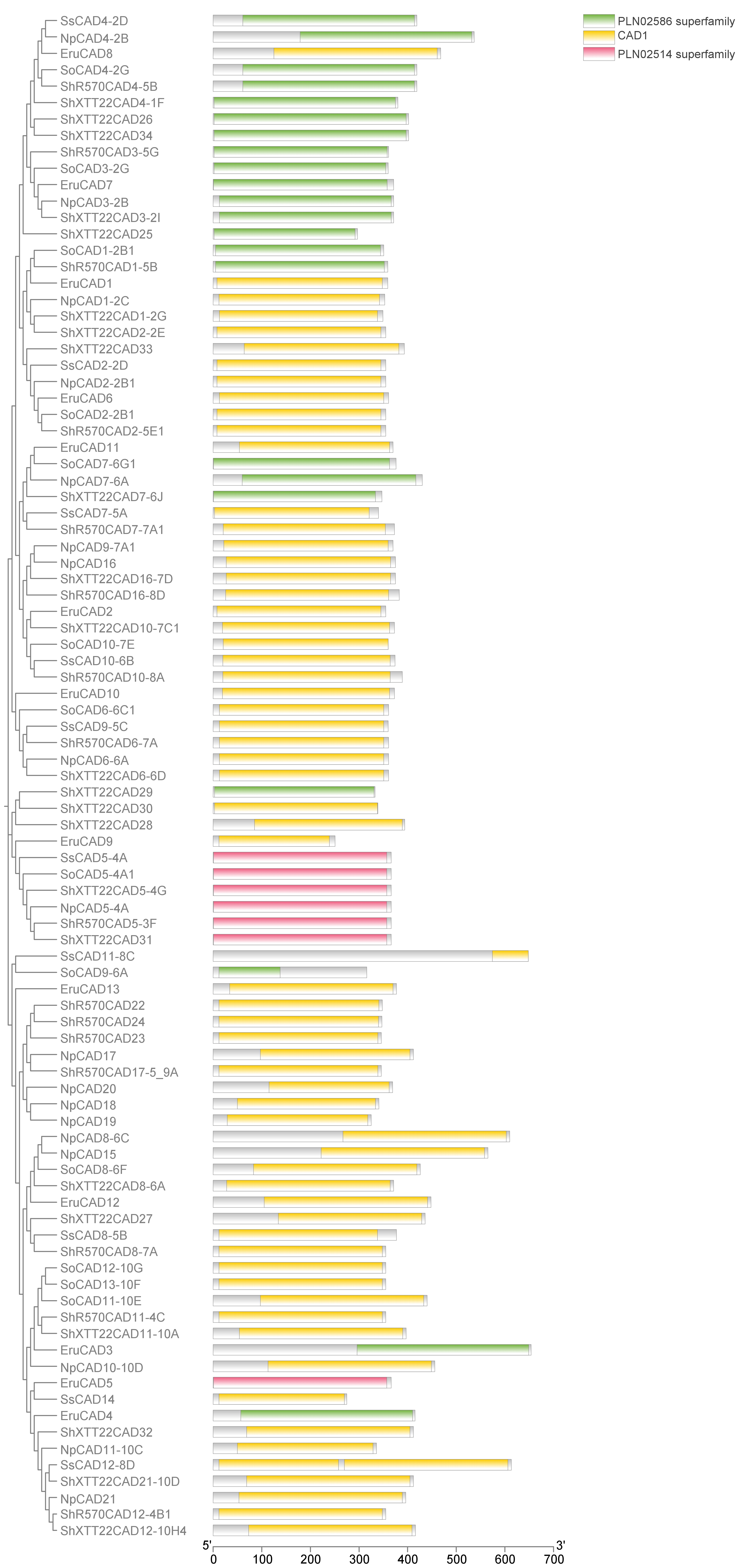

Supplement: Supplementary file 1 [file plants-14-02735-s001.zip › Figure S3.jpg]

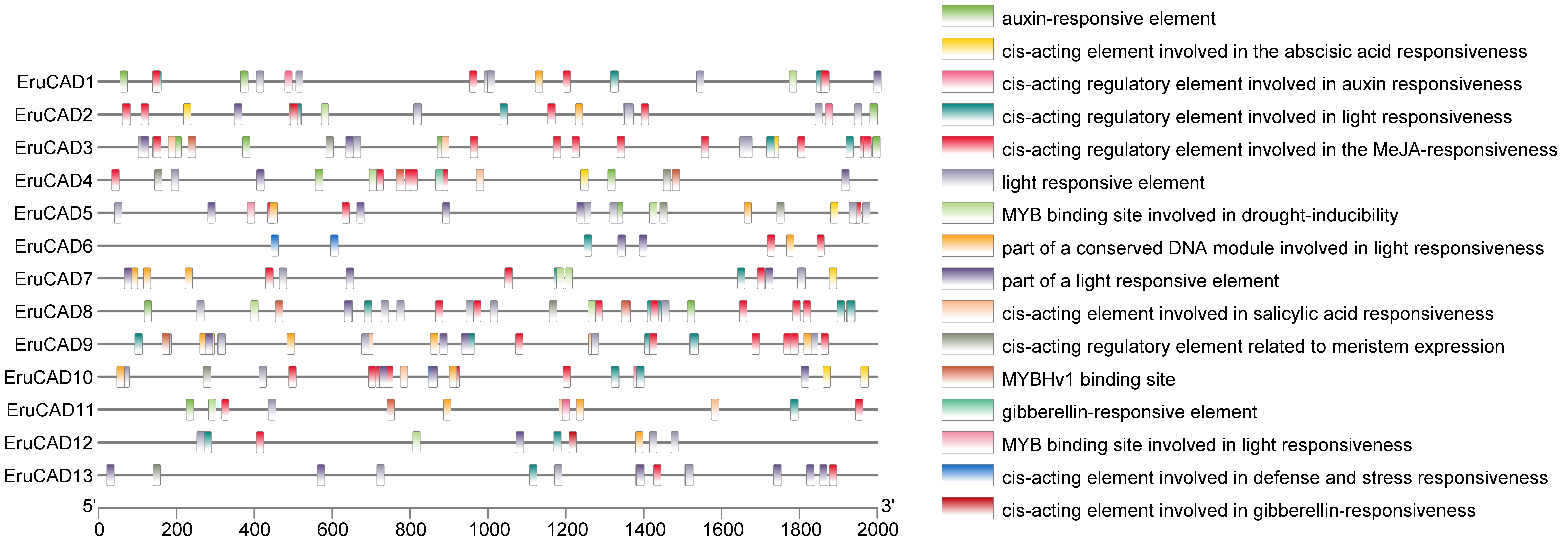

Supplement: Supplementary file 1 [file plants-14-02735-s001.zip › Figure S4/EruCAD.jpg]

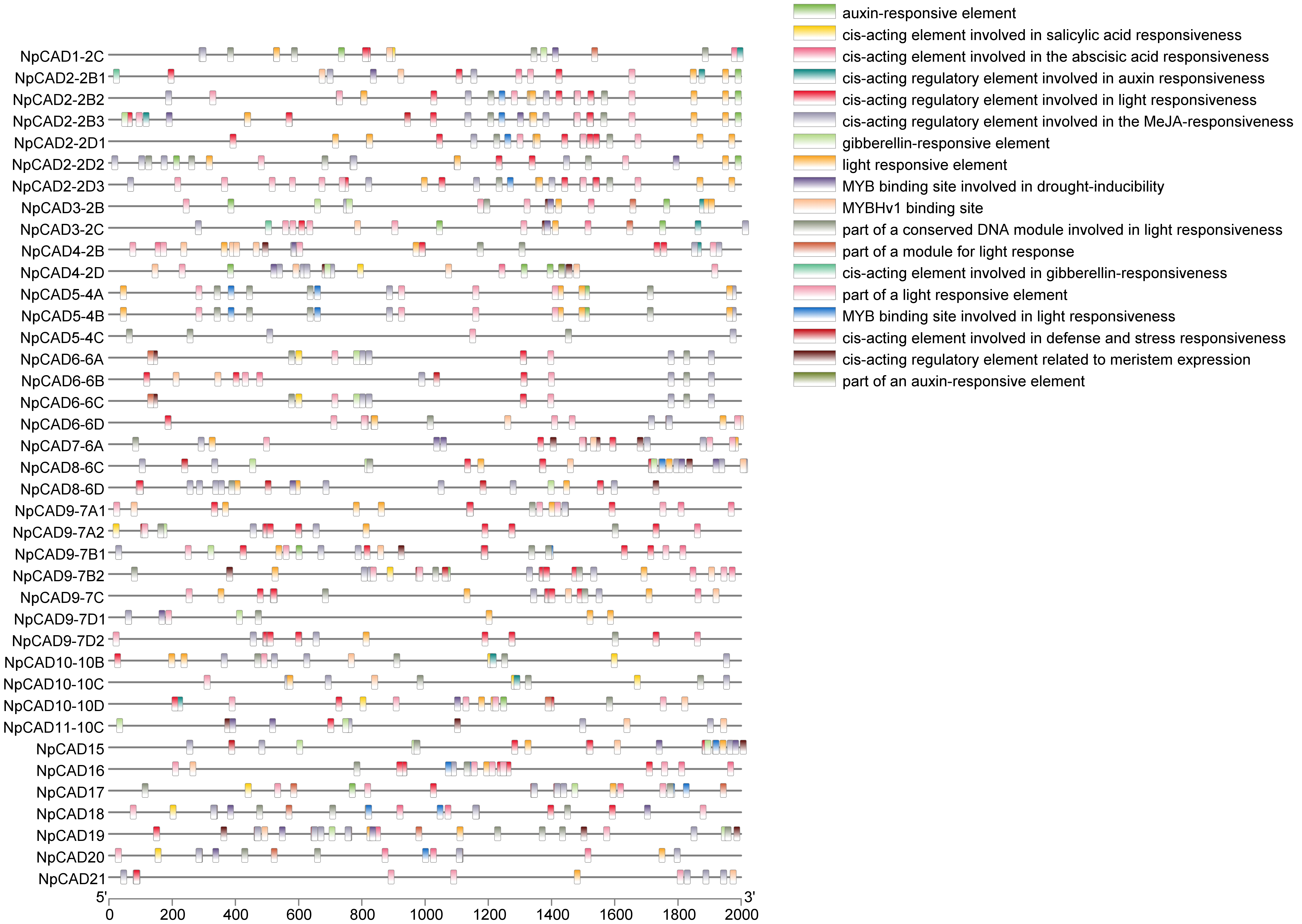

Supplement: Supplementary file 1 [file plants-14-02735-s001.zip › Figure S4/NpCAD.jpg]

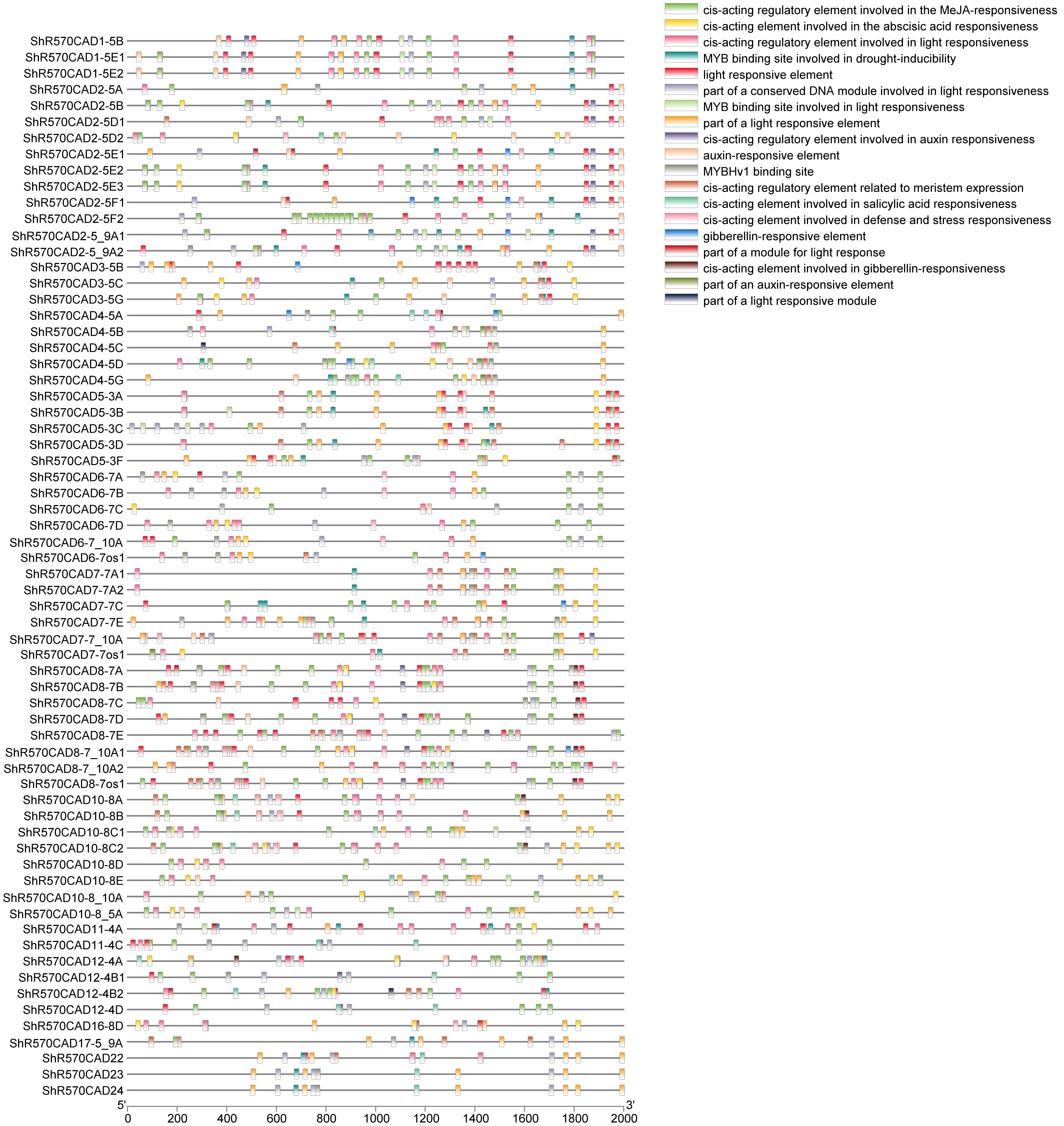

Supplement: Supplementary file 1 [file plants-14-02735-s001.zip › Figure S4/ShR570CAD.jpg]

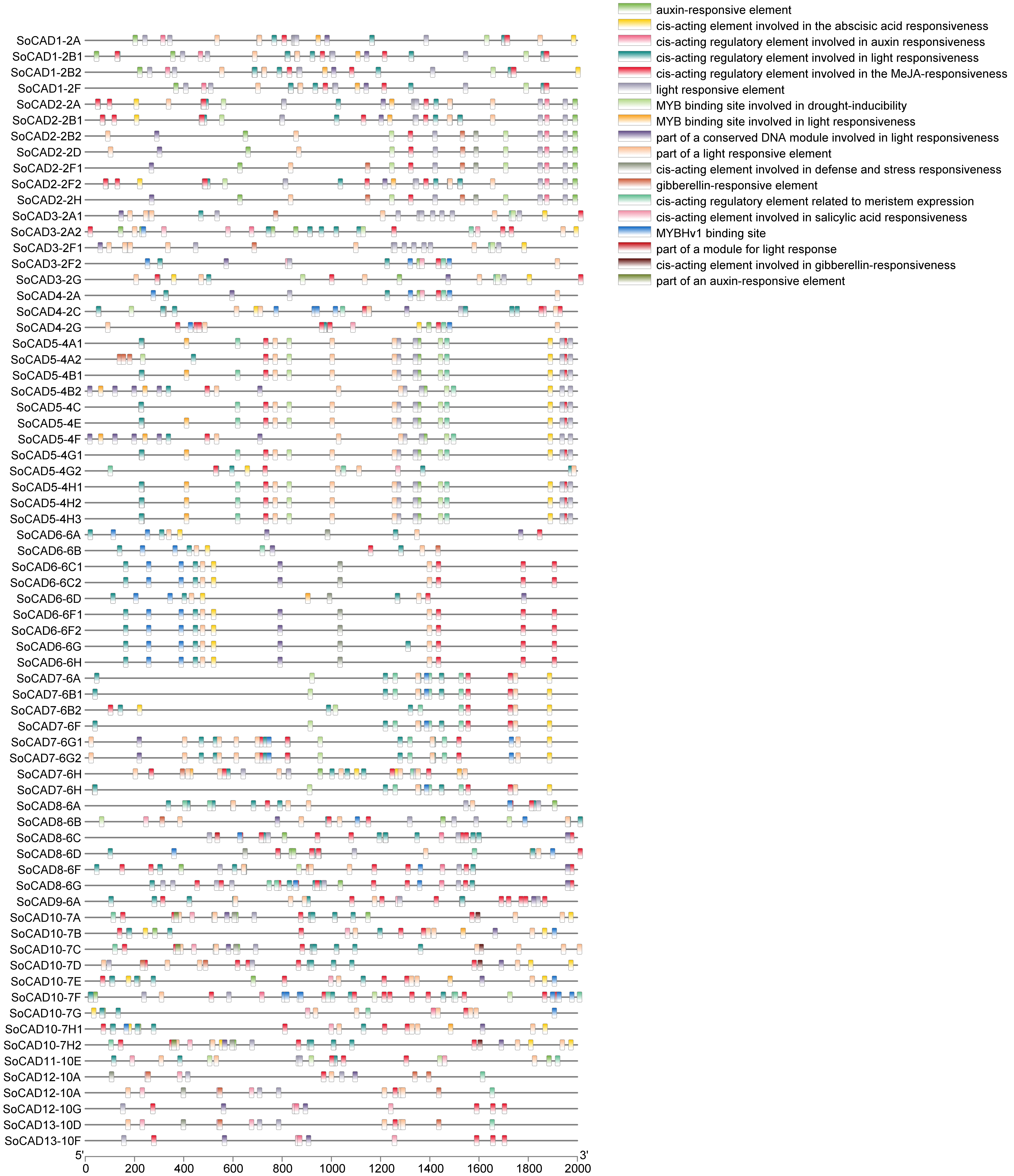

Supplement: Supplementary file 1 [file plants-14-02735-s001.zip › Figure S4/SoCAD.jpg]

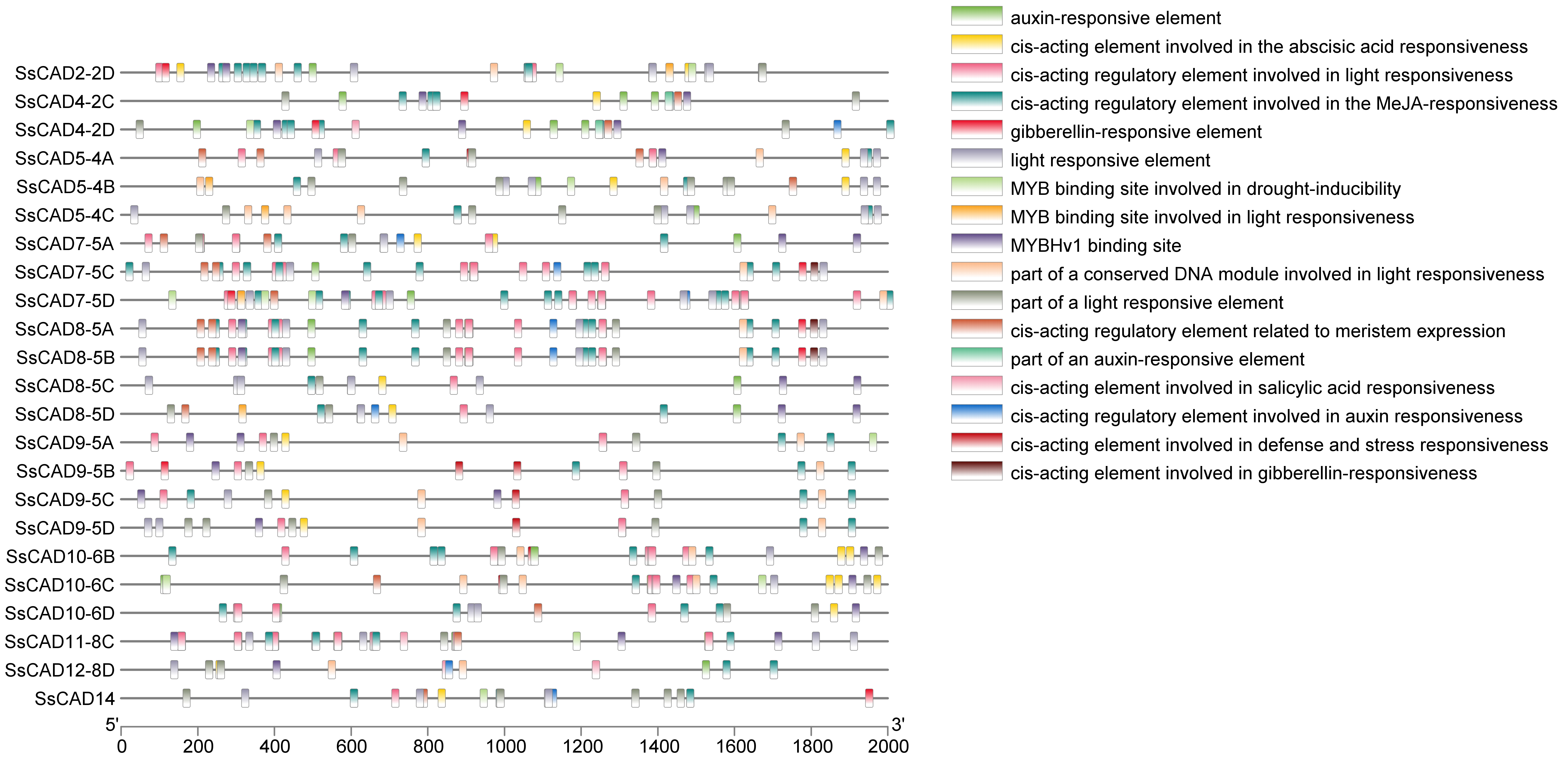

Supplement: Supplementary file 1 [file plants-14-02735-s001.zip › Figure S4/SsCAD.jpg]
